# Cerebral protection in aortic arch surgery: systematic review and meta-analysis

**DOI:** 10.1093/icvts/ivac128

**Published:** 2022-05-16

**Authors:** Djamila Abjigitova, Kevin M Veen, Gabriëlle van Tussenbroek, Mostafa M Mokhles, Jos A Bekkers, Johanna J M Takkenberg, Ad J J C Bogers

**Affiliations:** 1 Department of Cardiothoracic Surgery, Erasmus University Medical Center, Rotterdam, Netherlands; 2 Department of Cardiothoracic Surgery, Utrecht University Medical Center, Utrecht, Netherlands

**Keywords:** Cerebral perfusion, Bilateral, Unilateral, Antegrade, Retrograde, Deep hypothermic circulatory arrest

## Abstract

**Figure:**
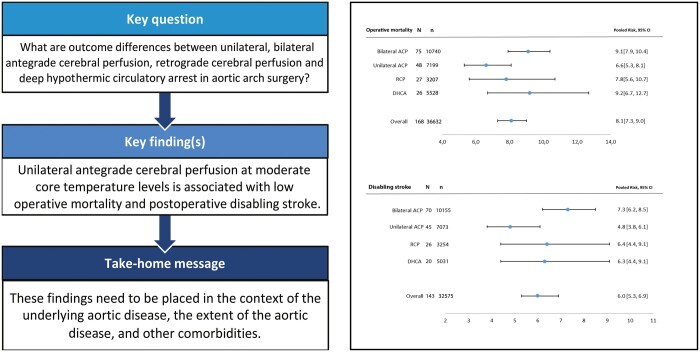

Consensus regarding optimal cerebral protection strategy in aortic arch surgery is lacking. We therefore performed a systematic review and meta-analysis to assess outcome differences between unilateral antegrade cerebral perfusion (ACP), bilateral ACP, retrograde cerebral perfusion (RCP) and deep hypothermic circulatory arrest (DHCA). A systematic literature search was performed in Embase, Medline, Web of Science, Cochrane and Google Scholar for all papers published till February 2021 reporting on early clinical outcome after aortic arch surgery utilizing either unilateral, bilateral ACP, RCP or DHCA. The primary outcome was operative mortality. Other key secondary endpoints were occurrence of postoperative disabling stroke, paraplegia, renal and respiratory failure. Pooled outcome risks were estimated using random-effects models. A total of 222 studies were included with a total of 43 720 patients. Pooled postoperative mortality in unilateral ACP group was 6.6% [95% confidence interval (CI) 5.3–8.1%], 9.1% (95% CI 7.9–10.4%), 7.8% (95% CI 5.6–10.7%), 9.2% (95% CI 6.7–12.7%) in bilateral ACP, RCP and DHCA groups, respectively. The incidence of postoperative disabling stroke was 4.8% (95% CI 3.8–6.1%) in the unilateral ACP group, 7.3% (95% CI 6.2–8.5%) in bilateral ACP, 6.4% (95% CI 4.4–9.1%) in RCP and 6.3% (95% CI 4.4–9.1%) in DHCA subgroups. The present meta-analysis summarizes the clinical outcomes of different cerebral protection techniques that have been used in clinical practice over the last decades. These outcomes may be used in advanced microsimulation model. These findings need to be placed in the context of the underlying aortic disease, the extent of the aortic disease and other comorbidities.

Prospero registration number: CRD42021246372

METC: MEC-2019-0825

## INTRODUCTION

Proximal aorta and arch replacements have shown a rapid growth in case volume since the evolvement of surgical techniques, advancement of circulatory management strategies and improvement of neuroprotective strategies in the last decades [1]. A substantial heterogeneity of cerebral protection techniques in aortic arch surgery between centres remains an issue [2]. Deep hypothermic circulatory arrest (DHCA) alone, or in conjunction with retrograde cerebral perfusion (RCP), or antegrade cerebral perfusion (ACP), unilateral or bilateral ACP are common. Furthermore, moderate and mild hypothermia has been increasingly utilized [3, 4]. The ideal method of cerebral protection remains undefined.

Last decade different pairwise and network meta-analyses were published to compare DHCA, RCP, bilateral and unilateral ACP, in different combinations [5–15]. Despite these important contributions, an overall clear presentation of the available data including all the available subgroups is lacking. Since there is no consensus, different cerebral protection techniques are being used for the same aortic procedures and pathologies. We, therefore, performed a systematic review and meta-analysis to assess outcome differences between unilateral ACP, bilateral ACP, RCP and DHCA to better inform clinical practice and future research.

## METHODS

### Protocol and registration

This systematic review was performed according to the checklist of the Preferred Reporting Items for Systematic Reviews and Meta-analysis (PRISMA) (Supplementary Material) for meta-analysis [16], and registered with PROSPERO (International Prospective Register of Systematic Reviews, CRD42021246372). The Medical Ethics Committee of Erasmus University Medical Center in Rotterdam granted approval for this study (MEC-2019-0825).

### Search strategy and selection criteria

On 17 February 2021, Embase, Medline, Web of Science, Cochrane and Google Scholar were searched by a biomedical information specialist (search terms are available in Supplementary Material). Two researchers (D.A. and G.T.) independently reviewed abstracts and full texts based on predefined inclusion and exclusion criteria. Observational, both retrospective and prospective, and randomized clinical trials (RCTs) that reported outcomes after aortic arch surgery in adults with a sample size ≥10 patients, published in English, were included. The aetiologies included were degenerative aortic aneurysms, acute type A aortic dissections and post-dissection chronic aneurysms. Non-original studies (reviews), case reports, poster publications, conference presentations, animal studies, editorials, studies not defining or incomplete reporting of outcome and data were excluded. The following additional, pre-specified, exclusion criteria were applied: studies reporting on hybrid aortic arch procedures, other than frozen elephant trunk, solely redo cases, type B aortic dissections, mini-sternotomy and all different approaches other than median sternotomy, articles on exclusively concomitant procedures. In case of disagreement, an agreement was negotiated till consensus was reached. In case of multiple publications on overlapping study populations, the largest series were included.

### Data extraction

Microsoft Office Excel 2016 (Microsoft Corp., Redmond, WA, USA) was used for data extraction. Two reviewers (D.A. and G.T.) independently performed data extraction and recorded all data with a standardized data-extraction form. Single-arm studies (one group reported) and 2-arm (2-group) studies with a within-study comparison of different cerebral perfusion techniques (unilateral ACP, bilateral ACP, RCP and solely circulatory arrest) were extracted separately. Disagreements were resolved through discussion or consensus involving a third investigator. Outcomes are considered early if they occur within 30 days of surgery or during the initial hospital admission. Extracted baseline characteristics and outcomes are provided in Supplementary Material, Table S1. Since all the studies were combined in a single-arm manner and there was no comparator, no bias assessment was performed.

### Outcome measures

The primary outcome for this meta-analysis was operative mortality. The secondary endpoints included postoperative disabling stroke, postoperative transient ischaemic attack, paraplegia, new-onset dialysis, acute kidney injury, resternotomy for bleeding or tamponade, respiratory failure (mechanical ventilation >48 h or reintubation), tracheostomy, mediastinitis, pacemaker placement, length of stay in intensive care unit and total length of hospital stay. All clinical endpoint outcomes were defined based on standards of reporting in open and endovascular aortic surgery (STORAGE) guidelines [17]. Temperature classification is consistent with the International Aortic Arch Surgery Study Group (IAASSG) consensus guideline regarding the nomenclature of hypothermia during aortic arch surgery [18].

### Statistical analysis

Inverse variance weighted pooled baseline patient and procedural characteristics were calculated. Outcomes were pooled on a logarithmic scale if the Shapiro–Wilk test revealed a skewed distribution. Outcomes were pooled in a random-effects model using the Der Simonian and Laird method to estimate the between-study variance [19]. In case an event was reported not to occurred, we assumed 0.5 patient experienced the event for pooling purposes (continuity correction). Heterogeneity was assessed using Cochrane Q and *I*^2^. Univariable meta-regression was conducted to explore potential causes of heterogeneity. The variables used to account for heterogeneity are sex, age, hypertension, diabetes mellitus, the presence of concomitant coronary artery disease, chronic obstructive pulmonary disease, history of cerebrovascular accident, presence of connective tissue disorder, history of cardiac surgery, type of pathology and presentation, extension of the aortic arch replacement, year of publication, type of cerebral protection, cardiopulmonary bypass (CPB) and aortic cross-clamp times, the lowest core temperature. Finally, we predefined 2 analytic subsets: patients who underwent aortic arch repair for degenerative aortic aneurysms, and those with acute aortic dissections. We aimed to summarize all the available data on different cerebral techniques and decided not to perform a formal comparison in the pre-determined statistical plan. No statistical comparisons were performed between different cerebral protection methods because the vast majority of included publications are observational single-arm analyses, with the differed inclusion period, clinical presentation and the complexity of the aortic disease.

## RESULTS

**Figure 1:**
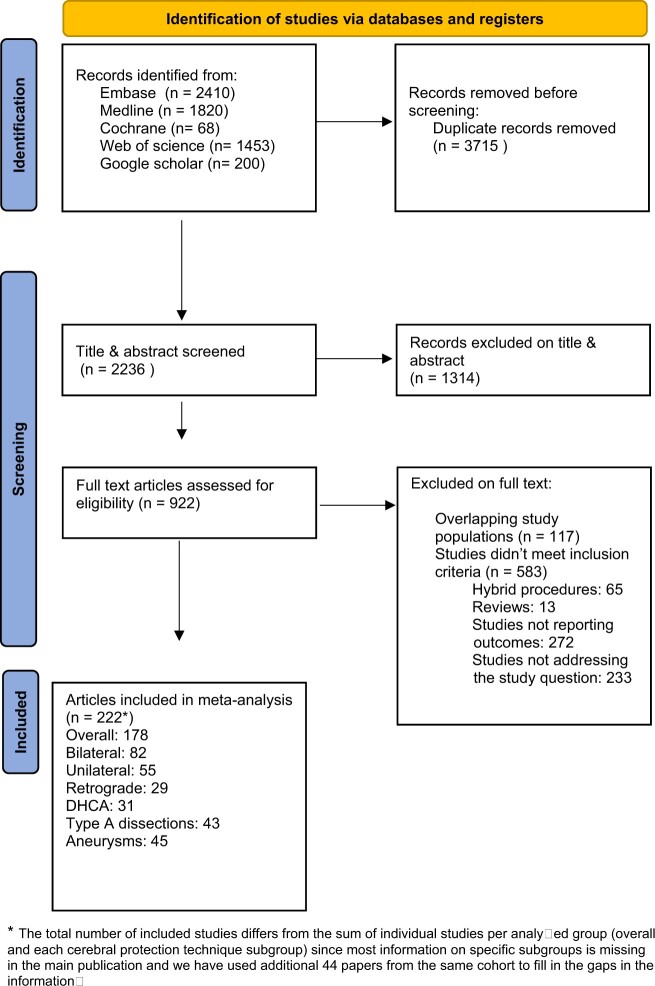
Preferred Reporting Items for Systematic Reviews and Meta-Analyses (PRISMA) flowchart of the analysis.

The literature search resulted in 5951 publications. After applying inclusion and exclusion criteria, 222 studies were included for the final analysis, of which 178 in the overall group, 82 in bilateral ACP, 55 publications were included in unilateral ACP, 29 in RCP and 31 in DHCA (Fig. 1). Two hundred and twenty-two studies were observational and 2 were RCTs.

### Study and patients characteristics

Individual study characteristics are presented in Supplementary Material, Table S2. In total, 37 275 patients with a mean age of 61.8 were included in the overall group. Pooled patient and procedural characteristics are presented in Table 1. Sixty per cent of the patients (60.0%) underwent hemiarch replacement, total aortic arch replacement was performed in 40.4% of all cases. In 59.2% of cases, patients presented with degenerative aortic aneurysm, in 17.6% of cases with post-chronic dissection aortic dilatation and in 38.1% with acute type A aortic dissection. The use of different cerebral perfusion techniques was evenly distributed in overall group (Table 1).

**Table 1:** Baseline characteristics

Characteristics	Overall		Bilateral ACP		Unilateral ACP		RCP		DHCA	
	Pooled estimate (CI)	*N* studies	Pooled estimate (CI)	*N* studies	Pooled estimate (CI)	*N* studies	Pooled estimate (CI)	*N* studies	Pooled estimate (CI)	*N* studies
Age	61.8 (61.7–61.9)	139	64.5 (64.3–64.8)	66	59.3 (59.1–59.5)	41	61.4 (61.0–61.9)	24	64.1 (63.8–64.5)	22
Male	66.7% (66.1–67.1)	173	68.9% (67.2–67.0)	80	69.8% (68.7–70.8)	54	67.3% (65.7–68.9)	28	63.4% (62.1–64.7)	28
Acute aortic dissection	38.1% (37.4–38.8)	174	35.9% (34.8–37.0)	80	51.6% (50.0–53.2)	53	38.5% (35.9–41.3)	29	29.0% (27.4–30.7)	30
Chronic dissection	17.6% (17.0–18.2)	174	18.5% (17.4–19.7)	80	19.6% (18.3–20.9)	51	9.4% (8.2–10.8)	29	20.6% (18.7–22.6)	30
Degenerative	59.2% (58.6–59.9)	175	57.0% (55.9–58.1)	80	49.1% (47.3–50.8)	54	65.2% (63.0–67.3)	29	62.6% (60.8–64.2)	30
Other	7.2% (6.8–7.7)	174	5.1% (4.4–5.9)	77	4.8% (4.1–5.6)	54	14.8% (12.4–17.5)	29	2.2% (1.7–2.9)	30
Hypertension	70.2% (70.0–71.0)	115	76.2% (75.1–77.2)	45	69.6% (68.4–70.7)	42	66.8% (64.7–68.9)	17	71.7% (70.3–73.0)	19
Emergency	36.8% (36.1–37.5)	90	33.5% (32.4–34.7)	44	36.4% (34.6–38.3)	24	44.3% (41.5–47.2)	18	34.6% (32.8–36.5)	13
History of CVA	10.9% (10.5–11.3)	102	14.6% (13.7–15.4)	51	6.4% (5.7–7.0)	28	9.6% (8.5–10.8)	18	11.1% (10.1–12.3)	16
Marfan	7.4% (7.0–7.9)	75	5.9% (5.2–6.6)	33	12.6% (11.4–13.9)	26	10.0% (8.3–12.0)	12	6.2% (5.2–7.3)	14
COPD	16.3% (15.8–16.8)	99	14.5% (13.6–15.4)	45	13.8% (12.7–14.9)	31	15.6% (13.9–17.5)	17	17.9% (16.7–19.2)	13
Previous heart surgery	17.9% (17.4–18.4)	105	16.3% (15.5–17.3)	50	13.7% (12.8–14.7)	31	18.1% (16.6–19.7)	19	21.8% (20.5–23.2)	17
CAD	21.4% (20.9–22.0)	80	21.6% (20.6–22.6)	42	17.1% (16.1–18.3)	22	28.0% (25.1–31.0)	9	23.9% (22.4–25.5)	11
DM	10.7% (10.3–11.1)	101	11.9% (11.1–12.8)	40	9.9% (9.2–10.7)	39	11.4% (10.0–13.0)	15	9.7% (8.7–10.8)	14
DHCA	37.1% (36.0–38.2)	172	0	0	0	0	0	0	100%	31
Unilateral ACP	38.5% (37.5–40.0)	171	0	0	100%	55	0	0	0	0
Bilateral ACP	49.7% (48.5–50.8)	173	100%	82	0	0	0	0	0	0
RCP	39.6% (38.4–40.9)	172	0	0	0	0	100%	0	0	0
CABG	16.6% (16.2–17.0)	120	15.9% (15.1–16.7)	56	12.8% (12.0–13.7)	35	20.7% (19.2–22.2)	24	19.2% (18.0–20.4)	21
Hemiarch replacement	60.0% (59.2–60.7)	173	37.0% (35.7–38.4)	80	46.3% (44.4–48.1)	52	63.5% (60.2–66.7)	29	76.6% (75.2–77.9)	29
Total arch replacement	40.4% (39.7–41.2)	174	62.1% (60.6–63.5)	80	53.5% (51.7–55.3)	52	34.7% (31.7–38.0)	29	28.5% (27.1–30.1)	29
Supra–coronary aortic replacement	63.8% (62.7–65.0)	35	63.4% (60.8–65.9)	13	55.1% (53.2–57.1)	11	54.0% (47.4–60.5)	6	65.0% (62.6–67.4)	5
Aortic root replacement	31.0% (30.4–31.7)	120	19.9% (18.9–20.9)	56	33.7% (32.5–34.9)	39	38.8% (36.8–40.8)	21	29.3% (27.7–31.1)	18
ET	13.6% (13.0–14.2)	178	15.5% (14.1–16.9)	82	15.8% (13.8–18.1)	55	6.6% (4.8–9.0)	29	10.6% (9.2–12.1)	31
FET	35.9% (34.2–37.6)	178	18.1% (16.3–20.0)	82	68.8% (66.5–70.9)	55	0.7% (0.4–1.2)	29	0	0
Lowest rectal temperature	24.7 (24.7–24.7)	68	25.8 (25.7–25.8)	30	25.8 (25.8–25.9)	23	20.4 (20.3–20.5)	10	20.7 (20.6–20.8)	11
Intraoperative outcome										
CPB time	200.7 (193.2–208.3)	136	217.6 (206.5–228.7)	69	188.8 (178.2–199.5)	43	198.6 (171.8–225.4)	21	170.9 (157.8–184.0)	22
ACC time	118.8 (113.9–123.8)	118	124.8 (117.8–131.8)	61	112.8 (106.6–119.0)	40	118.6 (102.6–134.5)	15	102.3 (93.6–110.9)	18
HCA time	38.2 (35.6–40.9)	85	48.0 (41.7–54.4)	41	27.6 (27.6–32.9)	26	32.2 (27.6–36.7)	18	23.1 (23.1–28.8)	21
Cerebral perfusion time	63.8 (52.7–75.0)	22								
ACP time	75.3 (70.1–80.6)	67	88.9 (80.7–97.0)	53	34.3 (30.2–38.3)	34				
RCP time	29.5 (23.9–35.1)	11					29.7 (21.8–37.7)	13		

ACC: aortic cross-clamp; ACP: antegrade cerebral perfusion; AF: atrial fibrillation; AKI: acute kidney insufficiency; CABG: coronary artery bypass grafting; CAD: coronary artery disease; CI: confidence interval; COPD: chronic obstructive pulmonary disease; CPB: cardiopulmonary bypass; CVA: cerebrovascular accident; DHCA: deep hypothermic circulatory arrest; DM: diabetes mellitus; ET: elephant trunk; FET: frozen elephant trunk; HCA: hypothermic circulatory arrest; ICU: intensive care unit; RCP: retrograde cerebral perfusion.

### Clinical outcomes

**Figure 2:**
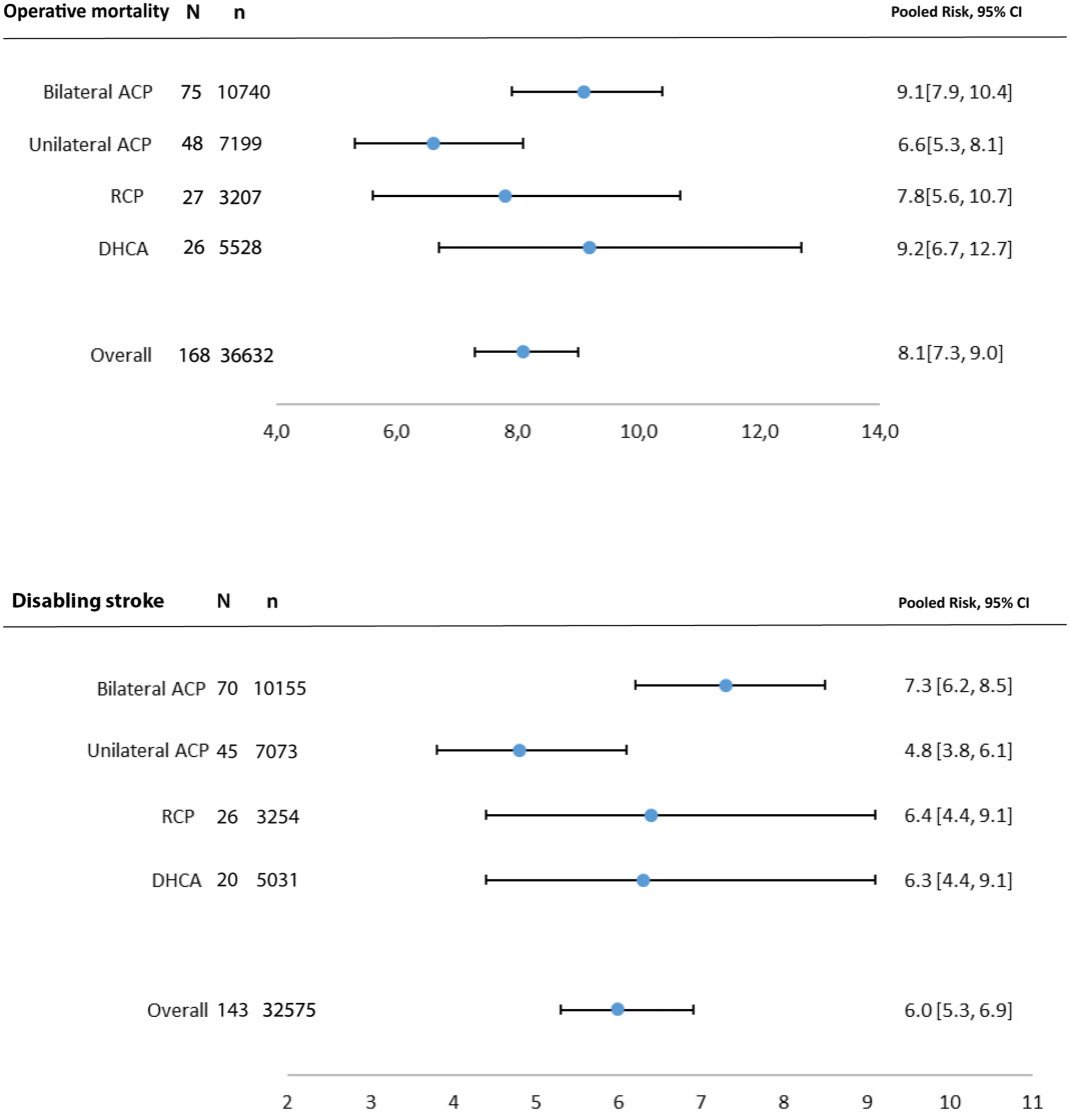
Forest plots of operative mortality and disabling stroke. The results are presented for the 4 cerebral perfusion techniques and an overall group. The results are expressed as pooled risk (PR) along with 95% confidence intervals (CIs). ACP: antegrade cerebral perfusion; CVA: cerebral vascular accident; DHCA: deep hypothermic circulatory arrest; *N*: number of studies; *n*: number of patients; RCP: retrograde cerebral perfusion.

The main results of the data synthesis are reported in Fig. 2. Operative mortality in overall group was 8.1% [95% confidence interval (CI) 7.3–9.0%]. The incidence of postoperative development of disabling stroke was 6.0% (95% CI 5.3–6.9%). Heterogeneity was high in all outcome measures (Table 2). Meta-regression identified several potential sources of heterogeneity. Especially, the indication acute aortic dissection was identified as major source of heterogeneity, as it explained 36% of the found heterogeneity. Furthermore, longer CPB time, lower mean core body temperature and earlier study period were associated with higher early mortality risk (Supplementary Material, Table S3). A lower age was associated with higher mortality. A *post-hoc* analysis revealed that studies with a lower age also had more cases of acute aortic dissection (Spearman *R*: -0.43).

**Table 2:** Outcome

Characteristic	Overall		Bilateral ACP		Unilateral ACP		RCP		DHCA	
	Pooled estimate	*N* studies (*I*^2^)	Pooled estimate	*N* studies (*I*^2^)	Pooled estimate	*N* studies (*I*^2^)	Pooled estimate	*N* studies (*I*^2^)	Pooled estimate	*N* studies (*I*^2^)
Operative mortality	8.1 (7.3–9.0)	168 (85.9%)	9.1 (7.9–10.4)	75 (75.8%)	6.6 (5.3–8.1)	48 (73.2%)	7.8 (5.6–10.7)	27 (80.4%)	9.2 (6.7–12.7)	26 (89.2%)
Disabling stroke	6.0 (5.3–6.9)	143 (86.4%)	7.3 (6.2–8.5)	70 (71.7%)	4.8 (3.8–6.1)	45 (68.4%)	6.4 (4.4–9.1)	26 (80.1%)	6.3 (4.4–9.1)	20 (86.7%)
TIA	7.7 (6.5–9.0)	105 (89.9%)	8.9 (7.3–10.9)	48 (83.1%)	7.4 (5.5–9.8)	32 (78.1%)	7.5 (4.3–13.0)	16 (88.2%)	5.7 (3.3–9.7)	18 (92.5%)
Paraplegia	2.4 (1.8–3.2)	53 (70.0%)	2.5 (1.8–3.6)	35 (51.0%)	2.4 (1.6–3.4)	13 (25.9%)	3.4 (1.1–10.1)	2 (0%)	4.7 (1.0–20.4)	2 (0%)
Resternotomy	8.3 (7.3–9.4)	106 (83.7%)	7.6 (6.2–9.3)	46 (75.2%)	6.8 (5.5–8.4)	37 (72.6%)	7.5 (4.9–11.3)	19 (84.6%)	7.0 (5.3–9.1)	17 (64.3%)
Dialysis	7.0 (5.7–8.6)	64 (91.3%)	6.8 (4.8–9.3)	28 (85.3%)	6.5 (4.5–9.4)	19 (88.5%)	6.1 (3.8–9.7)	10 (73.4%)	6.1 (2.5–14.3)	6 (95.1%)
Respiratory failure	17.2 (15.0–19.6)	88 (93.4%)	19.9 (16.3–24.1)	48 (93.3%)	14.6 (10.0–20.7)	27 (93.8%)	17.3 (12.5–23.6)	17 (88.5%)	12.9 (7.2–22.2)	10 (92.2%)
Tracheostoma	7.8 (6.0–10.2)	35 (89.3%)	9.0 (6.5–12.4)	16 (73.4%)	7.5 (3.9–13.9)	12 (92.3%)	5.4 (2.8–10.4)	6 (16.4%)	4.0 (2.0–7.7)	6 (88.1%)
New-onset AF	22.9 (15.5–32.6	12 (95.1%)	23.0 (16.0–31.8)	3 (0%)	35.9 (23.0–51.1)	2 (2.3%)	23.7 (7.5–54.4)	3 (77.2%	3.3 (0.2–36.7)	1 (NA)
AKI	10.8 (8.8–13.1)	64 (91.6%)	15.5 (11.0–21.2)	29 (92.6%)	9.7 (7.4–12.6)	24 (63.1%)	11.2 (7.6–16.4)	18 (90.0%)	0.8 (0.5–14.1)	6 (76.7%)
Mediastinitis	3.1 (2.4–4.0)	48 (71.0%)	3.8 (2.8–5.3)	25 (66.0%)	5.0 (3.5–7.3)	10 (0%)	1.9 (0.6–5.5)	6 (62.0%)	0.2 (0. 1–0.2)	4 (0%)
Pacemaker	6.8 (2.6–16.5)	9 (94.5%)	3.8 (2.3–6.1)	3 (0%)	4.6 (2.1–10.1)	6 (61.5%)	2.8 (0.7–10.5)	2 (0%)	0.5 (0.1–23.1)	2 (85.7%)
ICU stay (days)	5.7 (5.3–6.2)	44 (99.3%)	7.2 (6.0–8.5)	17 (95.8%)	5.2 (4.6–5.9)	20 (99.6%)	5.2 (3.3–7.0)	5 (94.4%)	5.9 (3.3–8.5)	4 (95.5%)
Hospital stay (days)	18.9 (17.0–20.8)	43 (98.9%)	25.2 (21.4–29.1)	17 (98.2%)	15.7 (12.3–19.1)	16 (99.0%)	13.9 (10.3–17.5)	8 (93.1%)	13.1 (10.7–15.6)	9 (98.0%

AF: atrial fibrillation; AKI: acute kidney insufficiency; ICU: intensive care unit; TIA: transient ischaemic attack.

Presence of preoperative acute aortic pathology was associated with a higher risk of postoperative disabling stroke (Supplementary Material, Table S4).

Sensitivity analysis did not reveal major changes in pooled outcomes when studies with a sample size lower than 25th percentile were temporarily excluded (Supplementary Material, Table S5).

Leave-one-out sensitivity analysis did not change the significance of all outcomes.

### Different cerebral perfusion cohorts

There were substantial differences in the baseline characteristics of cerebral perfusion cohorts. Patients presenting with acute aortic dissections were mainly treated with unilateral ACP (51.6%) and least utilizing DHCA (29.0%) (Table 1). The prevalence of Marfan connecting tissue disorder was highest in the unilateral ACP group (12.6%). Patients in the unilateral ACP group had the lowest prevalence of preoperative cerebrovascular accidents (6.4%); however, they had the highest prevalence of previous cardiac surgery (13.7%). Patients treated with RCP had the highest prevalence of coronary artery disease (28.0%).

Hemiarch replacement was mainly performed under DHCA (76.6%), whereas bilateral ACP was mainly utilized for total aortic arch replacements (62.1%) (Table 1). DHCA was least utilized for total aortic arch replacements (28.5%). Furthermore, the concomitant elephant trunk procedures were least performed utilizing RCP or DHCA, 6.6% and 10.6%, respectively. The majority of frozen elephant trunk procedures were performed utilizing the unilateral ACP (68.8%) and none with DHCA. The mean lowest core body temperature was comparable between the bilateral and unilateral ACP groups, mean 25.8°C (Table 2). However, RCP and DHCA had lower mean core body temperatures, mean 20.4°C and 20.7°C, respectively. The longest CPB, aortic cross-clamp, hypothermic circulatory arrest and selective cerebral perfusion times were noted in the bilateral ACP subgroup (Table 1).

Pooled postoperative mortality in unilateral ACP group was 6.6% (95% CI 5.3–8.1%) (Fig. 2). Furthermore, mortality rates were 9.1% (95% CI 7.9–10.4%), 7.8% (95% CI 5.6–10.7%) and 9.2% (95% CI 6.7–12.7%) in bilateral ACP, RCP and DHCA groups, respectively. The largest difference was noted in the incidence of postoperative disabling stroke between the subgroups. The lowest incidence of postoperative disabling stroke was seen in the unilateral ACP group, 4.8% (95% CI 3.8–6.1%) (Fig. 2). Risk of postoperative paraplegia in bilateral and unilateral ACP groups was 2.5% (95% CI 1.8–3.6%) and 2.4% (95% CI 1.6–3.4%), respectively. The highest paraplegia rate was noted in DHCA group, 4.7% (95% CI 1.0–20.4%) (Fig. 2).

Complete secondary outcomes of the 4 cerebral perfusion techniques are shown in Table 2.

### Type A aortic dissection and aneurysm cohorts

Forty-three studies were included in type A aortic dissection and 45 in aneurysm subgroups. Pooled patient and procedural characteristics of the type A aortic dissection and aneurysm subgroups are presented in Table 3. Patients presenting with acute type A aortic dissection were younger (mean age 49.8 years) in contrast to patients presenting with aneurysmal disease (mean age 63.9 years). Previous cardiac surgery was performed in 22.9% of all patients with chronic degenerative aneurysms and in 9.6% of patients with acute aortic dissections. Prevalence of coronary artery disease was 28.2% in patients with aneurysmal disease, and 13.4% in patients with acute aortic dissections. RCP and bilateral ACP were the main cerebral perfusion techniques utilized in patients with aneurysms, 72.2% and 60.1%, respectively. Utilization of DHCA, bilateral ACP, unilateral ACP and RCP among the patients with acute type A aortic dissections was equally distributed, 39.9%, 39.0%, 45.8%, 36.5%, respectively. Concomitant elephant trunk was performed in 16.6% of patients with aneurysmal disease, and only in 1.9% of patients with acute dissections.

**Table 3:** Baseline characteristics chronic aneurysms and acute dissections

Characteristics	Aneurysms		Acute dissections	
	Pooled estimate	*N* studies	Pooled estimate	*N* studies
Age	63.9 (63.7–64.2)	38	49.8 (49.5–50.1)	33
Male	68.1% (67.1–69.0)	44	63.7% (62.3–65.1)	41
Acute aortic dissection	0%	45	100%	43
Degenerative	100%	45	0%	43
Other	2.0% (1.6–2.5)	43	0.6% (0.4–0.9)	42
Hypertension	71.7% (70.6–72.8)	31	67.8% (66.2–69.4)	28
Emergency	14.0% (12.3–16.0)	11	56.4% (50.7–62.0)	6
History of CVA	13.1% (12.3–14.0)	29	8.0% (7.0–9.2)	20
Marfan	8.2% (7.2–9.3)	14	6.1% (5.2–7.1)	24
COPD	14.0% (13.2–14.8)	31	10.1% (8.9–11.5)	21
Previous heart surgery	22.4% (21.4–23.3)	29	9.6% (8.3–11.0)	17
CAD	28.2% (26.8–29.5)	19	13.4% (12.0–14.9)	18
DM	10.7% (10.0–11.4)	33	9.0% (8.0–10.1)	26
DHCA	15.6% (13.7–17.7)	45	39.9% (37.1–42.8)	43
Unilateral ACP	27.0% (25.0–29.1)	45	39.0% (36.4–41.8)	43
Bilateral ACP	60.1% (57.9–62.3)	45	45.8% (42.7–49.0)	43
RCP	72.2% (69.4–74.8)	45	36.5% (34.2–39.0)	43
CABG	18.7% (17.9–19.6)	36	9.6% (8.6–10.7)	25
Hemiarch replacement	69.8% (68.5–71.1)	45	62.0% (60.1–63.9)	42
Total arch replacement	30.2% (28.9–31.5)	45	34.9% (33.0–36.9)	42
Supra-coronary aortic replacement	44.8% (40.9–48.8)	8	50.5% (45.8–55.2)	8
Aortic root replacement	33.9% (32.6–35.2)	31	23.8% (22.4–25.3)	30
ET	16.6% (14.9–18.4)	44	1.9% (1.4–2.5)	42
FET	13.3% (11.5–15.4)	44	10.3% (8.1–13.1)	43
Lowest rectal temperature	25.9 (25.8–25.9)	18	24.7 (24.6–24.7)	15
Intraoperative outcome				
CPB time	194.0 (180.4–207.6)	35	211.1 (189.4–232.8)	34
ACC time	111.1 (102.6–119.7)	32	124.6 (113.5–135.6)	29
HCA time	35.0 (30.4–39.6)	20	35.5 (31.7–39.3)	25
Cerebral perfusion time			35.1 (33.2–36.9)	2
ACP time	74.7 (65.8–83.6)	26	78.0 (63.9–92.0)	15
RCP time			28.5 (0.31–56.7)	3

ACC: aortic cross-clamp; ACP: antegrade cerebral perfusion; AF: atrial fibrillation; AKI: acute kidney insufficiency; CABG: coronary artery bypass grafting; CAD: coronary artery disease; COPD: chronic obstructive pulmonary disease; CPB: cardiopulmonary bypass; CVA: cerebrovascular accident; DHCA: deep hypothermic circulatory arrest; DM: diabetes mellitus; ET: elephant trunk; FET: frozen elephant trunk; HCA: hypothermic circulatory arrest; ICU: intensive care unit; RCP: retrograde cerebral perfusion.

**Figure 3:**
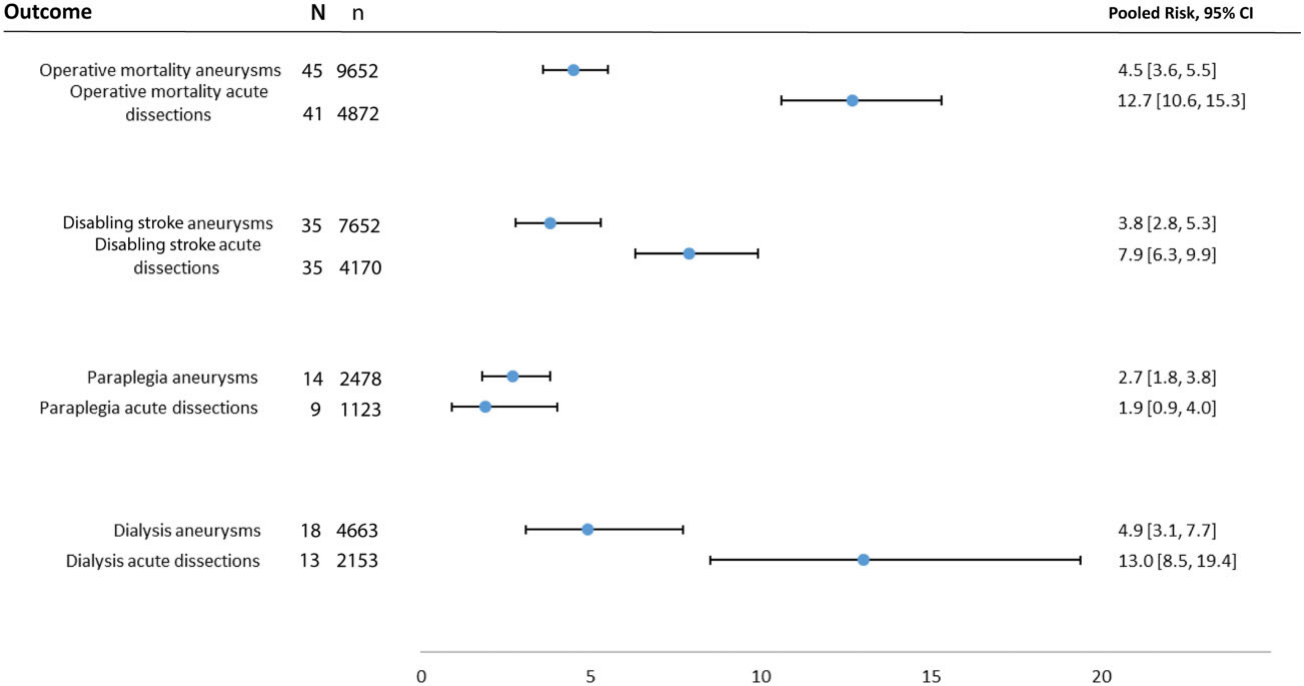
Forest plots of operative mortality, disabling stroke, paraplegia and dialysis outcomes for the subgroups aneurysm and acute aortic dissection. The results are expressed as pooled risk (PR) along with 95% confidence intervals (CIs). ACP: antegrade cerebral perfusion; CVA: cerebral vascular accident; DHCA, deep hypothermic circulatory arrest; *N*: number of studies; *n*: number of patients; RCP, retrograde cerebral perfusion.

Pooled operative mortality in type A acute aortic dissection subgroup was 12.7% (95% CI 10.6–5.3%), and 4.5% (95% CI 3.6–5.5%) in patients with aneurysmal disease. Pooled postoperative incidence of disabling stroke was 7.9% (95% CI 6.3–9.9%) in acute type A dissection subgroup, and 3.8% (95% CI 2.8–5.3%) in aneurysm subgroup (Fig. 3).

Meta-regression of moderators for these subgroups showed that the total replacement of aortic arch and use of bilateral ACP were associated with higher operative mortality rate in the aneurysm group (Supplementary Material, Table S7). The use of unilateral ACP in aneurysm subgroup was associated with lower incidence of disabling strokes, as opposed to bilateral ACP (Supplementary Material, Table S8).

For acute aortic dissections, total replacement of the aortic arch, the prolonged length of CPB time and the lower mean rectal body temperature were associated with higher operative mortality (Supplementary Material, Table S9). Presence of preoperative chronic obstructive pulmonary disease was associated with higher postoperative disabling stroke (Supplementary Material, Table S10).

## DISCUSSION

In this study, we provide a comprehensive systematic review of outcomes after hemiarch and aortic arch replacement surgery utilizing different cerebral protection strategies and 2 major aetiologies (acute aortic dissection and degenerative aneurysm) in light of evolved neuroprotective strategies and existent differences worldwide. To the best of our knowledge, this is the first comprehensive review and meta-analysis on the 4 different cerebral protection techniques including single-arm studies, extending beyond the existing data.

This study shows that the group of patients with unilateral ACP had lowest rate of operative mortality and disabling stroke. This is observed in spite of the unilateral ACP group having the highest proportion of acute aortic dissections, especially relative to the bilateral group. Moreover, the unilateral ACP group showed low paraplegia, dialysis rates and short length of intensive care unit stay.

However, it is important to note that the imbalance seen between the different cerebral perfusion groups in baseline variables may influence the outcomes, and could be found by chance. Furthermore, it is important to realize that not all patients might be found suitable for unilateral ACP because of their cerebral vascular anatomy and physiology. In addition, bilateral ACP is often selectively used during cases with anticipated prolonged circulatory arrest or when a significant drop in left-sided oximetry is observed. Hence the results of this single-arm meta-analysis should be seen and interpreted in the light of these differences.

In the past, several meta-analyses have compared unilateral ACP with bilateral ACP in pairwise comparisons. Angeloni and colleagues [5] found no difference in operative mortality (9.2% vs 8.6%, *P* = 0.78) and postoperative permanent neurologic dysfunction (6.5% vs 6.1%, *P* = 0.80) between bilateral and unilateral ACP. Also, Tian and colleagues [6] were unable to find any significant difference for operative mortality (9.9% vs 11.3%, *P* = 0.90) and permanent neurologic dysfunction (8.8% vs 10.6%, *P* = 0.85) between bilateral and unilateral ACP.

Furthermore, in our study, meta-regression in the aneurysm subgroup revealed that the unilateral ACP was associated with lower rates of postoperative disabling stroke, as opposed to bilateral ACP. In a recent study by Norton and colleagues [20], unilateral ACP showed favourable short- and mid-term results compared with bilateral ACP in patients with type A aortic dissections. Though the majority of patients receiving unilateral ACP underwent hemiarch replacement, in comparison with patients who underwent total aortic arch replacement where mainly bilateral ACP was used. Hence patients in bilateral ACP group were exposed to longer hypothermic circulatory arrest times.

Recent network meta-analysis by Hameed and colleagues [15] compared ACP, RCP and DHCA. They found that both ACP and RCP were associated with lower postoperative stroke and operative mortality compared with DHCA, with no difference in any outcome when comparing ACP and RCP. Contrary to our findings, they found that the use of unilateral or bilateral ACP did not affect the incidence of postoperative stroke. In line with our findings, arrest temperature was significant effect modifier of postoperative incidence of stroke. Specifically, in our study, the use of moderate and mild hypothermia in conjunction with unilateral ACP was associated with less disabling stroke. We also found that study period significantly correlated only with operative mortality in the overall and DHCA groups, with older studies reporting worse results.

Surgical society is aware of the existent differences between the centres in the utilization of cerebral perfusion techniques. Hence recent collaborative efforts led to the establishment of the International Aortic Arch Surgery Study Group (IAASSG) and ARCH registry to better evaluate patient outcomes after aortic arch surgery and achieve clinical consensus [1]. Certain restraint exists in the surgical community to adapt more evolved selective cerebral perfusion techniques and warmer hypothermic circulatory arrest temperatures. This is primary attributable to existent belief that this would result in not sufficient protection of contralateral hemisphere in patients with incomplete circle of Willis. Current expert consensus document of the European Association for Cardio-Thoracic Surgery and the European Society for Vascular Surgery does recommend preoperative imaging studies to assess the patency and morphology of the circle of Willis where treatment involves the aortic arch [17]. Though, it is still not standard care to perform a preoperative computed tomography angiography (CTA) of cerebral vessels to evaluate the patency of circle of Willis. Furthermore, different clinical studies were undertaken to assess the anatomical differences of the circle of Willis with CTA [21–23]. Anatomical variations of the circle of Willis are very common. Interestingly, these studies show that only 27% of the cases to have a complete polygon. However, despite these abnormalities, the patients developed no neurological deficits after the surgical procedure, which involved carotid clamping [24]. Furthermore, CTA faces technical limitations in the imaging of some arteries and diagnosis of hypoplasia and aplasia during the CTA will not be sufficiently reliable. The correlations between neurologic complications and circle of Willis variations detected by high-resolution CTA have not been published.

### Study limitations

This study shares the usual limitations of meta-analyses of observational studies. All the outcomes showed high heterogeneity. This might be the result of existing differences in the baseline characteristics, suggesting different underlying populations. Notwithstanding, we performed a robust meta-regression and identified several potential sources for the heterogeneity. The meta-regression analysis only adjusted for the publication date but not for the different timelines of surgical practice. The included studies often report very extensive recruitment period and no statistical method exists to adjust for this except individual patient data.

Additionally, heterogeneity may arise due to different definitions for stroke, as well as sample size and surgical expertise. Due to multiple testing, some found differences may be due to chance. Furthermore, publication bias may be present which can potentially lead to underestimation of the estimates. We did not assess publication bias using funnel plots, as they do not allow for meaningful interpretation in case of absolute risk outcomes because of substantial methodological limitations, which may lead to funnel plot asymmetry [25].

The aim of this research is to provide readers with comprehensive overview on clinical outcomes after aortic arch surgery utilizing different cerebral protection methods. Therefore, it is not meant to make any comparisons and extreme caution is needed for interpretation of the results in the light of known limitations.

Two RCTs, both single-blinded were included in this meta-analysis. We were unable to perform a sensitivity analysis and pool the outcome data of RCTs since only one study reported primary outcome.

During the extraction process of the data, we have encountered a relative paucity on the reported data regarding the preoperative evaluation of circle of Willis. Therefore, we are unable to report whether the patients who received unilateral ACP, had preoperative evaluation of their cerebral vessels, including the completeness of circle of Willis.

## CONCLUSIONS

The present meta-analysis summarizes the clinical outcomes of different cerebral protection techniques that have been used in clinical practice over the last decades. These findings need to be placed in the context of the underlying aortic disease, the extent of the aortic disease and comorbidities. Thus, further global multi-institutional collaboration is warranted to provide better-powered analyses and further formulate and validate risk predictor models.

## SUPPLEMENTARY MATERIAL

Supplementary material is available at *ICVTS* online.

## Supplementary Material

ivac128_Supplementary_Data
