# Supplementary material for: Cerebral protection in aortic arch surgery: systematic review and meta-analysis
Source: Interact Cardiovasc Thorac Surg. 2022 May 16;35(3):ivac128. doi: 10.1093/icvts/ivac128 (PMC9419700; doi:10.1093/icvts/ivac128)
Supplement: ivac128_Supplementary_Data [file ivac128_supplementary_data.docx]

**Supplementary Table 1:** Extracted outcome and baseline variables

| **Baseline variables** | **Outcomes** |
| --- | --- |
| Age | CPB time |
| Male | ACC time |
| Acute aortic dissection | HCA time |
| Chronic dissection | Cerebral perfusion time |
| Degenerative | ACP time |
| Other | RCP time |
| Hypertension | Operative mortality |
| History of CVA | Disabling stroke |
| Marfan | TIA |
| COPD | Paraplegia |
| Previous heart surgery | Restornotomy |
| CAD | Dialysis |
| DM | Respiratory failure |
| No cerebral perfusion | Tracheostoma |
| Unilateral ACP | New onset AF |
| Bilateral ACP | AKI |
| RCP | Mediastinitis |
| CABG | Pacemaker |
| Hemiarch replacement | ICU stay |
| Total arch replacement | Hospital stay |
| Supra-coronary aortic replacement |  |
| Aortic root replacement |  |
| ET |  |
| FET |  |
| Lowest rectal temperature |  |

ACC, aortic cross clamp; ACP, antegrade cerebral perfusion; AF, atrial fibrillation; AKI, acute kidney insufficiency; CABG, coronary artery bypass grafting; CAD, coronary artery disease; COPD, chronic obstructive pulmonary disease; CPB, cardiopulmonary bypass; CVA cerebrovascular accident; DM, diabetes mellitus; ET, elephant trunk; FET, frozen elephant trunk; HCA, hypothermic circulatory arrest; ICU, intensive care unit; RCP, retrograde cerebral perfusion; TIA, transient ischemic attack.

**Supplementary Table 2:** Baseline characteristics of individual studies

| **Author** | **Type of study** | **N** | **Age** | **Male**  **(n,%)** | **Acute dissection**  **(n, %)** | **Chronic dissection**  **(n, %)** | **Degenerative (n, %)** | **BiACP**  **(n, %)** | **UniACP**  **(n, %)** | **RCP**  **(n, %)** | **DHCA**  **(n, %)** |
| --- | --- | --- | --- | --- | --- | --- | --- | --- | --- | --- | --- |
| **Ito (2017)[1]** | Observational | 49 | 74 | 38 | 3(6.1) | 13(26.5) | 33(67.3) | 49(100) | 0(0) | 0(0) | 0(0) |
| **Abdelgawad (2017) [2]** | Observational | 43 | 59.2 | 38 | 13(30.2) | 0(0) | 32(74.4) | 0(0) | 18(41.9) | 25(58.1) | 0(0) |
| **Abjigitova (2019) [3]** | Observational | 172 | 64 | 92 | 58(33.7) | 19(11) | 84(48.8) | 133(77.3) | 0(0) | 2(1.2) | 37(21.5) |
| **Akpinar (2001) [4]** | Observational | 26 | 71 | 19 | 0(0) | 1(3.8) | 25(96.2) | 0(0) | 0(0) | 0(0) | 26(100) |
| **Alamanni (1995) [5]** | Observational | 35 | 60.3 | 26 | 17(48.6) | 0(0) | 18(51.4) | 16(45.7) | 0(0) | 0(0) | 19(54.3) |
| **Al-Sabri (2019)[6]** | Observational | 107 | 62.1 | 66 | 90(84.1) | 0(0) | 30(28) | 70(65.4) | 0(0) | 0(0) | 37(34.6) |
| **Amano (2019)[7]** | Observational | 191 | 65 | 95 | 191(100) | 0(0) | 0(0) | 191(100) | 0(0) | 0(0) | 0(0) |
| **Ando (1994) [8]** | Observational | 42 | 50.4 | 28 | 19(45.2) | 23(54.8) | 0(0) | 42(100) | 0(0) | 0(0) | 0(0) |
| **Apaydin (2009) [9]** | Observational | 161 | 55 | 113 | 137(85.1) | 0(0) | 24(14.9) | 19(11.8) | 0(0) | 94(58.4) | 48(29.8) |
| **Apostolakis (2008) [10]** | Observational | 48 | 60.5 | 36 | 48(100) | 0(0) | 0(0) | 5(10.4) | 18(37.5) | 25(52.1) | 0(0) |
| **Arnaoutakis (2016)[11]** | Observational | 589 | 60.7 | 412 | 0(0) | 0(0) | 589(100) | 0(0) | 118(20) | 471(80) | 0(0) |
| **Aytekin (2016)[12]** | Observational | 30 | 56.50 | 17 | 0(0) | 0(0) | 30(100) | 0(0) | 30(100) | 0(0) | 0(0) |
| **Bachet (1999) [13]** | Observational | 171 | 56.5 | 118 | 49(28.7) | 0(0) | 122(71.3) | (0) | 171(100) | (0) | (0) |
| **Bachet (2002)[14]** | Observational | 206 | 56 | 142 | 54(26.2) | 46(22.3) | 106(51.5) | 206(100) | 0(0) | 0(0) | 0(0) |
| **Banarjee (2014)[15]** | Observational | 90 | 62 | 68 | 90(100) | 0(0) | 0(0) | 90(100) | 0(0) | 0(0) | 0(0) |
| **Bashir (2014)[16]** | Observational | 287 | 63.7 | 172 | 0(0) | 0(0) | 147(51.2) | 0(0) | (0) | 140(48.8) | 0(0) |
| **Beckmann (2020) [17]** | Observational | 211 | 59.2 | 139 | 96(45.5) | 47(22.3) | 68(32.2) | 211(100) | 0(0) | 0(0) | 0(0) |
| **Bjurbom (2014) [18]** | Observational | 29 | 66.5 | 19 | (0) | (0) | (0) | 29(100) | 0(0) | 0(0) | 0(0) |
| **Brat (2015)[19]** | Observational | 30 | 60 | 17 | 0(0) | 0(0) | 30(100) | 23(76.7) | 0(0) | 0(0) | 7(23.3) |
| **Casthely (1985) [20]** | Observational | 17 |  | 13 | 2(11.8) | 0(0) | 15(88.2) | 0(0) | 0(0) | 0(0) | 17(100) |
| **Cefarelli (2017) [21]** | Observational | 791 | 63 | 456 | 0(0) | 72(9.1) | 672(85) | 636(80.4) | 0(0) | 0(0) | 155(19.6) |
| **Centofanti (2016) [22]** | Observational | 333 | 64 | 243 | 190(57.1) | 0(0) | 81(24.3) | 142(42.6) | 129(38.7) | 27(8.1) | 35(10.5) |
| **Chen (2010) [23]** | Observational | 28 | 51.2 | 22 | 27(96.4) | 1(3.6) | 0(0) | 28(100) | 0(0) | 0(0) | 0(0) |
| **Chen (2018) [24]** | Observational | 100 | 49.5 | 78 | 100(100) | 0(0) | 0(0) | 0(0) | 0(0) | 0(0) | 100(100) |
| **Chen (2018)[25]** | Observational | 68 | 58 | 57 | 68(100) | 0(0) | 0(0) | 65(95.6) | 0(0) | 3(4.4) | 0(0) |
| **Cheng (2018) [26]** | RCT | 65 | 51.3 | 45 | 65(100) | 0(0) | 0(0) | 0(0) | 65(100) | 0(0) | 0(0) |
| **Cho (2008)[27]** | Observational | 28 | 54 | 14 | 28(100) | 0(0) | 0(0) | 25(89.3) | 0(0) | 2(7.1) | 1(3.6) |
| **Chung (2019)[28]** | Observational | 1653 | 62 |  | (0) | (0) | (0) | 0(0) | 1233(74.6) | 80(4.8) | 340(20.6) |
| **Colli (2016)[29]** | Observational | 185 | 63 | 105 | 185(100) | 0(0) | 0(0) | 67(36.2) | 0(0) | 101(54.6) | 17(9.2) |
| **Cook (2006)[30]** | Observational | 72 | 65.9 | 38 | 10(13.9) | 12(16.7) | 40(55.6) | (0) | (0) | 0(0) | 0(0) |
| **Dai (2015)[31]** | Observational | 41 | 49.1 | 25 | 41(100) | 0(0) | 0(0) | (0) | 41(100) | (0) | (0) |
| **Damberg (2017)[32]** | Observational | 613 | 63.7 | 375 | 36(5.9) | 44(7.2) | 520(84.8) | 0(0) | 0(0) | 0(0) | 613(100) |
| **Danner (2007)[33]** | Observational | 20 | 57.2 | 10 | 20(100) | 0(0) | 0(0) | 0(0) | 0(0) | 0(0) | 20(100) |
| **Davies (2011)[34]** | Observational | 75 | 57.5 | 56 | 22(29.3) | 13(17.3) | 54(72) | 53(70.7) | 0(0) | 10(13.3) | 6(8) |
| **Detter (2019)[35]** | Observational | 62 | 64.5 | 38 | 23(37.1) | 19(30.6) | 20(32.3) | 62(100) | 0(0) | 0(0) | 0(0) |
| **Deville (1988)[36]** | Observational | 41 | 55.8 | 24 | 30(73.2) | 0(0) | 11(26.8) | 0(0) | 0(0) | 0(0) | 41(100) |
| **Di Eusanio (2007) [37]** | Observational | 55 | 60 | 33 | 2(3.6) | 3(5.5) | 49(89.1) | 0(0) | 0(0) | 0(0) | 55(100) |
| **Di Eusanio (2002)[38]** | Observational | 413 | 63 | 268 | 116(28.1) | 70(16.9) | 227(55) | 413(100) | 0(0) | 0(0) | 0(0) |
| **Dong (2020) [39]** | Observational | 61 | 50.1 | 50 | 61(100) | 0(0) | 0(0) | 30(49.2) | 36(59) | 0(0) | 0(0) |
| **Ehrlich (1997)[40]** | Observational | 143 | 55 | 99 | 78(54.5) | 23(16.1) | 42(29.4) | 0(0) | 0(0) | 0(0) | 143(100) |
| **Eldeiry (2018)[41]** | Observational | 206 | 60.2 | 56 | (0) | (0) | (0) | 0(0) | 206(100) | 0(0) | 0(0) |
| **Estrera (2008)[42]** | Observational | 1107 | 64 | 682 | 279(25.2) | 204(18.4) | 624(56.4) | (0) | (0) | 907(81.9) | (0) |
| **Farhat (2007)[43]** | Observational | 15 | 61 | 10 | 15(100) | 0(0) | 0(0) | 15(100) | 0(0) | 0(0) | 0(0) |
| **Ganapathi (2014)[44]** | Observational | 440 | 57.0 | 300 | 91(20.7) | 34(7.7) | 315(71.6) | 0(0) | 360(81.8) | 80(18.2) | 0(0) |
| **Gatti (2017)[45]** | Observational | 344 | 66.9 | 256 | 91(26.5) | 2(0.6) | 237(68.9) | 0(0) | 0(0) | 344(100) | 0(0) |
| **Ghincea (2019)[46]** | Observational | 295 | 58.8 | 216 | 82(27.8) | 0(0) | 213(72.2) | 0(0) | 295(100) | 0(0) | 0(0) |
| **Guo (2014)[47]** | Observational | 16 | 50.0 | 12 | 16(100) | 0(0) | 0(0) | 0(0) | 16(100) | 0(0) | 0(0) |
| **Hata (2018)[48]** | Observational | 187 | 67.4 | 106 | 187(100) | 0(0) | 0(0) | 0(0) | 0(0) | 57(30.5) | 130(69.5) |
| **Hirano (2020)[49]** | Observational | 115 | 68.6 | 99 | 0(0) | 38(33) | 77(67) | 115(100) | 0(0) | 0(0) | 0(0) |
| **Hiraoka (2014)[50]** | Observational | 140 | 73.1 | 96 | 38(27.1) | 0(0) | 42(30) | 140(100) | 0(0) | 0(0) | 0(0) |
| **Ho (2020)[51]** | Observational | 41 | 60 | 34 | 31(75.6) | 13(31.7) | 9(22) | 41(100) | 0(0) | 0(0) | 0(0) |
| **Ianfrancesco (2015)[52]** | Observational | 58 | 76 | 20 | 8(13.8) | 9(15.5) | 33(56.9) | 54(93.1) | 0(0) | 0(0) | 4(6.9) |
| **Iba (2013)[53]** | Observational | 1007 | 72 | 668 | 230(22.8) | 0(0) | 777(77.2) | 1007(100) | (0) | (0) | (0) |
| **Imasaka (2017)[54]** | Observational | 200 | 71 | 132 | 0(0) | 0(0) | 200(100) | 200(100) | 0(0) | 0(0) | 0(0) |
| **Immer (2008)[55]** | Observational | 567 | 61.6 | 414 | 247(43.6) | 0(0) | 320(56.4) | 91(16) | 89(15.7) | 0(0) | 387(68.3) |
| **Inamura (2006) [56]** | Observational | 38 | 59.3 | 17 | 38(100) | 0(0) | 0(0) | 0(0) | 38(100) | 0(0) | 0(0) |
| **Jabagi (2021) [57]** | Observational | 66 | 67 | 43 | 0(0) | 1(1.5) | 65(98.5) | 0(0) | 66(100) | 0(0) | 0(0) |
| **Jacobs (2001) [58]** | Observational | 50 | 47 | 38 | 0(0) | 3(6) | 36(72) | 50(100) | 0(0) | 0(0) | 0(0) |
| **Kaku (2014)[59]** | Observational | 107 | 70 | 88 | 12(11.2) | 0(0) | 90(84.1) | 107(100) | 0(0) | 0(0) | 0(0) |
| **Kamenskaya (2017) [60]** | RCT | 58 | 53.1 | 45 | 0(0) | 58(100) | 0(0) | 0(0) | 29(50) | 0(0) | 29(50) |
| **Kan (2006) [61]** | Observational | 23 | 61.0 | 11 | 23(100) | 0(0) | 0(0) | 0(0) | 1(4.3) | 22(95.7) | 0(0) |
| **Kaneda (2005) [62]** | Observational | 68 | 68.2 | 34 | 50(73.5) | 0(0) | 18(26.5) | 51(75) | 0(0) | 17(25) | 0(0) |
| **Kaneko (2014) [63]** | Observational | 467 | 61.8 | 319 | 48(10.3) | 10(2.1) | 409(87.6) | 0(0) | 114(24.4) | 77(16.5) | 276(59.1) |
| **Kasama (2019) [64]** | Observational | 48 | 80 | 40 | 0(0) | 7(14.6) | 40(83.3) | 48(100) | 0(0) | 0(0) | 0(0) |
| **Kazui (2002) [65]** | Observational | 330 | 62.9 | 220 | 89(27) | 77(23.3) | 164(49.7) | 330(100) | 0(0) | 0(0) | 0(0) |
| **Kazui (2000) [66]** | Observational | 70 | 65 | 47 | 70(100) | 0(0) | 0(0) | 70(100) | 0(0) | 0(0) | 0(0) |
| **Khaladj (2008) [67]** | Observational | 501 | 64 | 320 | 153(30.5) | 23(4.6) | 256(51.1) | 501(100) | 0(0) | 0(0) | 0(0) |
| **Khullar (2017)[68]** | Observational | 567 | 62.9 | 374 | 98(17.3) | 0(0) | 415(73.2) | 144(25.4) | 0(0) | 65(11.5) | 335(59.1) |
| **Kim (2011) [69]** | Observational | 36 | 61.9 | 19 | 31(86.1) | 0(0) | 5(13.9) | 0(0) | 36(100) | 0(0) | 0(0) |
| **Kim (2018) [70]** | Observational | 232 | 62.5 | 123 | 84(36.2) | 27(11.6) | 123(53) | 0(0) | 67(28.9) | 0(0) | 165(71.1) |
| **Kim (2018) [71]** | Observational | 99 | 59 | 50 | 99(100) | 0(0) | 0(0) | 78(78.8) | 0(0) | 16(16.2) | 0(0) |
| **Kirali (2002) [72]** | Observational | 56 | 49.3 | 50 | 38(67.9) | 18(32.1) | 0(0) | 0(0) | 0(0) | 56(100) | 0(0) |
| **Kozlov (2018) [73]** | Observational | 37 | 54.7 | 24 | 11(29.7) | 26(70.3) | 0(0) | 0(0) | 37(100) | 0(0) | 0(0) |
| **Kremer (2019) [74]** | Observational | 68 | 61.8 | 48 | 34(50) | 0(0) | 34(50) | 68(100) | 0(0) | 0(0) | 0(0) |
| **Kucuker (2005) [75]** | Observational | 181 | 58 | 132 | 24(13.3) | 88(48.6) | 69(38.1) | 0(0) | 181(100) | 0(0) | 0(0) |
| **Kunihara (2005) [76]** | Observational | 273 | 62 | 164 | 105(38.5) | 0(0) | 168(61.5) | 0(0) | 0(0) | 6(2.2) | 273(100) |
| **Lakew (2005) [77]** | Observational | 327 | 60.5 | 227 | 0(0) | 0(0) | 327(100) | 0(0) | 0(0) | 0(0) | 327(100) |
| **Lau (2018) [78]** | Observational | 1043 | 65.7 | 639 | 374(35.9) | 0(0) | 669(64.1) | 0(0) | 0(0) | 50(4.8) | 993(95.2) |
| **Lee (2009) [79]** | Observational | 104 | 58.4 | 53 | 71(68.3) | 0(0) | 33(31.7) | 0(0) | 104(100) | 0(0) | 0(0) |
| **Legras (2013) [80]** | Observational | 63 | 61.8 | 46 | 63(100) | 0(0) | 0(0) | 0(0) | 54(85.7) | 0(0) | 9(14.3) |
| **Lei (2009) [81]** | Observational | 298 | 44.9 | 223 | (0) | (0) | 12(4) | 0(0) | 298(100) | 0(0) | 0(0) |
| **Leontyev (2019) [82]** | Observational | 925 | 62.3 | 585 | 0(0) | 69(7.5) | 761(82.3) | 517(55.9) | 184(19.9) | 0(0) | 224(24.2) |
| **Leshnower (2012) [83]** | Observational | 500 | 57.8 | 341 | 142(28.4) | 0(0) | 358(71.6) | 0(0) | 500(100) | 0(0) | 0(0) |
| **Li (2017) [84]** | Observational | 77 | 49.9 | 55 | 55(71.4) | 17(22.1) | 5(6.5) | 37(48.1) | 40(51.9) | 0(0) | 0(0) |
| **Liu (2020) [85]** | Observational | 300 | 53.8 | 237 | 300(100) | 0(0) | 0(0) | 0(0) | 300(100) | 0(0) | 0(0) |
| **Lopez Almodovar (2018) [86]** | Observational | 12 | 57.11 | 10 | 12(100) | 0(0) | 0(0) | 12(100) | 0(0) | 0(0) | 0(0) |
| **Lu (2012) [87]** | Observational | 263 | 51.4 | 200 | 243(92.4) | 20(7.6) | 0(0) | 128(48.7) | 135(51.3) | 0(0) | 0(0) |
| **Lytle (1995) [88]** | Observational | 43 | 64.3 | 28 | 24(55.8) | 0(0) | 19(44.2) | 0(0) | 0(0) | 43(100) | 0(0) |
| **Ma (2015) [89]** | Observational | 99 | 48.2 | 84 | 99(100) | 0(0) | 0(0) | 12(12.1) | 87(87.9) | 0(0) | 0(0) |
| **Ma (2018) [90]** | Observational | 62 | 47.15 | 50 | 52(83.9) | 10(16.1) | 0(0) | 62(100) | 0(0) | 0(0) | 0(0) |
| **Malaisrie (2015) [91]** | Observational | 177 | 57.8 | 138 | 0(0) | 0(0) | 177(100) | 0(0) | 19(10.7) | 158(89.3) | 0(0) |
| **Malvindi (2016) [92]** | Observational | 109 | 63 | 71 | 109(100) | 0(0) | 0(0) | 0(0) | 28(25.7) | 24(22) | 57(52.3) |
| **Maroto (2020) [93]** | Observational | 56 | 63 | 37 | 18(32.1) | 8(14.3) | 27(48.2) | 47(83.9) | 9(16.1) | 0(0) | 0(0) |
| **Matalanis (2003) [94]** | Observational | 62 | 64.6 | 49 | 32(51.6) | 22(35.5) | 0(0) | 25(40.3) | 0(0) | 23(37.1) | 14(22.6) |
| **Matsuyama (2011) [95]** | Observational | 186 | 68 | 144 | 79(42.5) | 9(4.8) | 98(52.7) | 119(64) | 0(0) | 67(36) | 0(0) |
| **Matsuzaki (2016) [96]** | Observational | 66 | 69.3 | 51 | 7(10.6) | 0(0) | 59(89.4) | 66(100) | 0(0) | 0(0) | 0(0) |
| **Matt (2017) [97]** | Observational | 141 | 62 | 96 | 141(100) | 0(0) | 0(0) | 0(0) | 141(100) | 0(0) | 0(0) |
| **Milewski (2010) [98]** | Observational | 776 | 60.4 | 527 | 0(0) | 73(9.4) | 688(88.7) | 94(12.1) | 0(0) | 682(87.9) | 0(0) |
| **Minakawa (2010)[99]** | Observational | 122 | 65 | 86 | 68(55.7) | 0(0) | 54(44.3) | 122(100) | 0(0) | 0(0) | 0(0) |
| **Miyamoto (2018) [100]** | Observational | 31 | 71.4 | 17 | 0(0) | 0(0) | 31(100) | 31(100) | 0(0) | 0(0) | 0(0) |
| **Mori (2003) [101]** | Observational | 27 | 61 | 15 | 22(81.5) | 5(18.5) | 0(0) | 27(100) | 0(0) | 0(0) | 0(0) |
| **Murzi (2014)[102]** | Observational | 221 | 64 | 146 | 114(51.6) | 0(0) | 107(48.4) | 132(59.7) | 0(0) | 37(16.7) | 18(8.1) |
| **Nakamura (2005) [103]** | Observational | 60 | 68.1 | 47 | 0(0) | 25(41.7) | 34(56.7) | 60(100) | 0(0) | 0(0) | 0(0) |
| **Nakamura (2011) [104]** | Observational | 143 | 70.4 | 100 | 14(9.8) | 30(21) | 97(67.8) | 0(0) | 0(0) | 143(100) | 0(0) |
| **Nakamura (2020) [105]** | Observational | 208 | 68 | 130 | 76(36.5) | 15(7.2) | 111(53.4) | 208(100) | 0(0) | 0(0) | 0(0) |
| **Numata (2013) [106]** | Observational | 243 | 68 | 155 | 99(40.7) | 0(0) | 144(59.3) | 243(100) | 0(0) | 0(0) | 0(0) |
| **Numata (2009) [107]** | Observational | 21 | 58 |  | 7(33.3) | 0(0) | 13(61.9) | 0(0) | 21(100) | 0(0) | 0(0) |
| **Numata (2003) [108]** | Observational | 120 | 69 | 99 | 16(13.3) | 16(13.3) | 79(65.8) | 0(0) | 120(100) | 0(0) | 0(0) |
| **Ochiai (2005) [109]** | Observational | 46 | 61.8 | 24 | 46(100) | 0(0) | 0(0) | 46(100) | (0) | (0) | (0) |
| **Ogino (2008) [110]** | Observational | 531 | 72 | 363 | 164(30.9) | 0(0) | 367(69.1) | 531(100) | 0(0) | 0(0) | 0(0) |
| **Ogino (2001) [111]** | Observational | 28 | 61.8 | 13 | 28(100) | 0(0) | 0(0) | 0(0) | 0(0) | 28(100) | 0(0) |
| **Ohata (2003) [112]** | Observational | 32 | 68.2 |  | 0(0) | 3(9.4) | 28(87.5) | 32(100) | 0(0) | 0(0) | 0(0) |
| **Ohtsubo (2002) [113]** | Observational | 47 | 68.5 | 20 | 47(100) | 0(0) | 0(0) | 29(61.7) | 0(0) | 0(0) | 18(38.3) |
| **Okada (2012) [114]** | Observational | 190 | 71.3 | 151 | 0(0) | 46(24.2) | 136(71.6) | 190(100) | 0(0) | 0(0) | 0(0) |
| **Okita (2013) [115]** | Observational | 423 | 69.3 | 313 | 81(19.1) | 72(17) | 270(63.8) | 423(100) | 0(0) | 0(0) | 0(0) |
| **Olsson (2006) [116]** | Observational | 65 | 59 | 49 | 41(63.1) | 0(0) | 24(36.9) | 48(73.8) | 17(26.2) | 0(0) | 0(0) |
| **Orlov (2020) [117]** | Observational | 75 | 63 | 40 | 14(18.7) | 25(33.3) | 33(44) | 75(100) | 0(0) | 0(0) | 0(0) |
| **Ozerdem (2010) [118]** | Observational | 82 | 59.5 | 38 | 28(34.1) | 0(0) | 46(56.1) | 0(0) | 82(100) | 0(0) | 0(0) |
| **Pacini (2015) [119]** | Observational | 641 | 62.9 | 447 | 300(46.8) | 0(0) | 297(46.3) | 641(100) | 0(0) | 0(0) | 0(0) |
| **Pagni (2013)[120]** | Observational | 132 | 59.8 | 88 | 132(100) | 0(0) | 0(0) | 0(0) | 50(37.9) | 75(56.8) | 3(2.3) |
| **Panos (2006) [121]** | Observational | 25 | 62.6 | 17 | 25(100) | 0(0) | 0(0) | 0(0) | 25(100) | 0(0) | 0(0) |
| **Park (2018) [122]** | Observational | 138 | 60.1 | 75 | 62(44.9) | 69(50) | 0(0) | 0(0) | 45(32.6) | 0(0) | 93(67.4) |
| **Patel (2011) [123]** | Observational | 721 | 59.3 | 497 | 284(39.4) | 0(0) | 416(57.7) | 400(55.5) | 0(0) | 641(88.9) | 0(0) |
| **Perera (2013) [124]** | Observational | 43 | 64 | 27 | 15(34.9) | 4(9.3) | 24(55.8) | 0(0) | 43(100) | 0(0) | 0(0) |
| **Perreas (2016) [125]** | Observational | 248 | 63.05 | 186 | 126(50.8) | 0(0) | 122(49.2) | 11(4.4) | 38(15.3) | 199(80.2) | 0(0) |
| **Preventza (2018) [126]** | Observational | 938 | 60.6 | 644 | 0(0) | 231(24.6) | 707(75.4) | 619(66) | 319(34) | 0(0) | 0(0) |
| **Qian (2013) [127]** | Observational | 54 | 45.7 | 43 | 54(100) | 0(0) | 0(0) | 0(0) | 54(100) | 0(0) | 0(0) |
| **Rungatscher (2016) [128]** | Observational | 210 | 64.6 | 141 | 85(40.5) | 0(0) | 125(59.5) | 87(41.4) | 0(0) | 0(0) | 123(58.6) |
| **Salem (2020) [129]** | Observational | 905 | 66.7 | 604 | 0(0) | 0(0) | 905(100) | (0) | (0) | (0) | 905(100) |
| **Salem (2020) [130]** | Observational | 339 | 63.7 | 219 | 339(100) | 0(0) | 0(0) | 339(100) | (0) | (0) | (0) |
| **Samanidis (2017) [131]** | Observational | 45 | 58 | 34 | 45(100) | 0(0) | 0(0) | 45(100) | 0(0) | 0(0) | 0(0) |
| **Schneider (2014) [132]** | Observational | 20 | 66 | 10 | 6(30) | 4(20) | 8(40) | 0(0) | 20(100) | 0(0) | 0(0) |
| **Senanayake (2012)** | Observational | 27 | 59 | 16 | 2(7.4) | 0(0) | 25(92.6) | 0(0) | 27(100) | 0(0) | 0(0) |
| **Shen (2018)** | Observational | 89 | 47.5 | 72 | 89(100) | 0(0) | 0(0) | 0(0) | 89(100) | 0(0) | 0(0) |
| **Shi (2011)** | Observational | 46 | 52.7 | 35 | 46(100) | 0(0) | 0(0) | 0(0) | 46(100) | 0(0) | 0(0) |
| **Shiiya (2000)** | Observational | 52 | 70 | 42 | 0(0) | 0(0) | 52(100) | 52(100) | 0(0) | 0(0) | 0(0) |
| **Shimamura (2008) [133]** | Observational | 126 | 67.8 | 86 | 31(24.6) | 26(20.6) | 69(54.8) | 126(100) | 0(0) | 0(0) | 0(0) |
| **Shimazaki (2004) [134]** | Observational | 39 | 72 | 27 | 0(0) | 10(25.6) | 29(74.4) | 39(100) | 0(0) | 0(0) | 0(0) |
| **Shimizu (2013) [135]** | Observational | 203 | 67.9 | 159 | 0(0) | 69(34) | 134(66) | 203(100) | 0(0) | 0(0) | 0(0) |
| **Shrestha (2014) [136]** | Observational | 103 | 59.4 | 77 | 74(71.8) | 0(0) | 29(28.2) | 103(100) | 0(0) | 0(0) | 0(0) |
| **Sinastra (2001) [137]** | Observational | 41 | 59.8 | 26 | 41(100) | 0(0) | 0(0) | 0(0) | 23(56.1) | 18(43.9) | 0(0) |
| **Spielvogel (2007) [138]** | Observational | 150 | 63 | 91 | 0(0) | 56(37.3) | 90(60) | 0(0) | 150(100) | 0(0) | 0(0) |
| **Stowe (1998) [139]** | Observational | 117 | 61.4 | 67 | 17(14.5) | 11(9.4) | 82(70.1) | 0(0) | 0(0) | 117(100) | 0(0) |
| **Suda (1996) [140]** | Observational | 24 | 64.2 |  | 23(95.8) | 0(0) | 0(0) | 0(0) | 0(0) | 0(0) | 24(100) |
| **Sultan (2018) [141]** | Observational | 248 | 61 | 160 | 0(0) | 18(7.3) | 230(92.7) | 0(0) | 0(0) | 248(100) | 0(0) |
| **Sundt (2008) [142]** | Observational | 347 | 65.7 | 195 | 60(17.3) | 62(17.9) | 195(56.2) | 0(0) | 74(21.3) | 53(15.3) | 220(63.4) |
| **Sundt (2004) [143]** | Observational | 19 | 68 | 8 | 1(5.3) | 6(31.6) | 12(63.2) | 19(100) | 0(0) | 10(52.6) | 0(0) |
| **Svensson (1993) [144]** | Observational | 656 | 64 | 373 | 77(11.7) | 199(30.3) | 316(48.2) | 0(0) | 0(0) | 0(0) | 656(100) |
| **Tabayashi (1993) [145]** | Observational | 20 | 65 | 6 | 20(100) | 0(0) | 0(0) | 17(85) | 0(0) | 0(0) | 3(15) |
| **Takahara (2003) [146]** | Observational | 100 | 64,6 | 66 | 40(40) | 11(11) | 49(49) | 100(100) | 0(0) | 0(0) | 0(0) |
| **Takano (2000)** | Observational | 59 | 67.3 | 39 | 0(0) | 17(28.8) | 42(71.2) | 59(100) | 0(0) | 0(0) | 0(0) |
| **Takashima (2014) [147]** | Observational | 123 | 73.9 | 25 | 0(0) | 12(9.8) | 111(90.2) | 123(100) | 0(0) | 0(0) | 0(0) |
| **Takayama (2009) [148]** | Observational | 136 | 60 | 107 | 0(0) | 16(11.8) | 106(77.9) | 0(0) | 136(100) | 0(0) | 0(0) |
| **Tan (1989) [149]** | Observational | 12 | 61.5 | 5 | 7(58.3) | 5(41.7) | 0(0) | 0(0) | 0(0) | 0(0) | 12(100) |
| **Tang (2013) [150]** | Observational | 101 | 65.2 |  | 101(100) | 0(0) | 0(0) | 0(0) | 0(0) | 0(0) | 101(100) |
| **Taniguchi (2007) [151]** | Observational | 52 | 70 | 40 | 0(0) | 11(21.2) | 36(69.2) | 52(100) | 0(0) | 0(0) | 0(0) |
| **Tokuda (2020) [152]** | Observational | 254 | 68.3 | 195 | 0(0) | 0(0) | 254(100) | 249(98) | 0(0) | 5(2) | 0(0) |
| **Tong (2017) [153]** | Observational | 203 | 49.4 | 128 | 203(100) | 0(0) | 0(0) | 121(59.6) | 82(40.4) | 0(0) | 0(0) |
| **Toyama (2009) [154]** | Observational | 26 | 68.9 | 14 | 16(61.5) | 1(3.8) | 9(34.6) | 26(100) | 0(0) | 0(0) | 0(0) |
| **Tsagakis (2010) [155]** | Observational | 68 | 58 | 52 | 68(100) | 0(0) | 0(0) | 68(100) | 0(0) | 0(0) | 0(0) |
| **Uchida (2010)[156]** | Observational | 122 | 69.7 | 81 | 66(54.1) | 0(0) | 56(45.9) | 122(100) | 0(0) | 0(0) | 0(0) |
| **Ueda (2003) [157]** | Observational | 103 | 65 | 77 | 14(13.6) | 21(20.4) | 68(66) | 103(100) | 0(0) | 0(0) | 0(0) |
| **Unal (2014) [158]** | Observational | 32 | 48.69 | 24 | 14(43.8) | 0(0) | 18(56.3) | 0(0) | 32(100) | 0(0) | 0(0) |
| **Urbanksi (2019) [159]** | Observational | 1000 | 63 | 700 | 0(0) | 41(4.1) | 940(94) | 0(0) | 1000(100) | 0(0) | 0(0) |
| **Usui (1999) [160]** | Observational | 166 | 60.2 | 93 | 166(100) | 0(0) | 0(0) | 91(54.8) | 0(0) | 75(45.2) | 0(0) |
| **Wai Sang (2020) [161]** | Observational | 42 | 65 | 31 | 0(0) | 0(0) | 42(100) | 0(0) | 42(100) | 0(0) | 0(0) |
| **Wang (2020) [162]** | Observational | 1708 | 47 | 1282 | 1166(68.3) | 381(22.3) | 161(9.4) | 0(0) | 1708(100) | 0(0) | 0(0) |
| **Watanabe (2011) [163]** | Observational | 50 | 60.6 | 34 | 28(56) | 0(0) | 22(44) | 50(100) | 0(0) | 0(0) | 0(0) |
| **Wiedemann (2013) [164]** | Observational | 329 | 59.1 | 220 | 329(100) | 0(0) | 0(0) | 38(11.6) | 53(16.1) | 0(0) | 116(35.3) |
| **Wu (2017) [165]** | Observational | 109 | 51.9 | 61 | 103(94.5) | 8(7.3) | 0(0) | 0(0) | 109(100) | 0(0) | 0(0) |
| **Wu (2020) [166]** | Observational | 69 | 50.4 | 55 | 69(100) | 0(0) | 0(0) | 69(100) | 0(0) | 0(0) | 0(0) |
| **Yamamoto (2014) [167]** | Observational | 12 | 66 | 4 | 12(100) | 0(0) | 0(0) | 0(0) | 12(100) | 0(0) | 0(0) |
| **Yilmazkaya (2014) [168]** | Observational | 44 | 55.2 | 32 | 6(13.6) | 0(0) | 38(86.4) | 0(0) | 44(100) | 0(0) | 0(0) |
| **Yu (2020) [169]** | Observational | 271 | 51.0 | 220 | 271(100) | 0(0) | 0(0) | 271(100) | 0(0) | 0(0) | 0(0) |
| **Zheng (2019) [170]** | Observational | 377 |  | 296 | 318(84.4) | 0(0) | 28(7.4) | 0(0) | 377(100) | 0(0) | 0(0) |
| **Zierer (2012) [171]** | Observational | 426 | 64 | 287 | 173(40.6) | 0(0) | 231(54.2) | 426(100) | 0(0) | 0(0) | 0(0) |
| **Zierer (2014)[172]** | Observational | 492 | 65.5 | 320 | 0(0) | 0(0) | 492(100) | 246(50) | 246(50) | 0(0) | 0(0) |
| **Zierer (2007) [173]** | Observational | 125 | 63 | 76 | 125(100) | 0(0) | 0(0) | 0(0) | 0(0) | 56(44.8) | 69(55.2) |
| **Shihata^1^**  **(2011) [174]** | Observational | 46 | 62 | 29 (63) | 0(0) | 17(37) | 29(63) | 0(0) | 46(100) | 0(0) | 0(0) |
| **Yilmazkaya^1^ (2014) [168]** | Observational | 44 | 55.2 | 32 (72,7) | 6(13,6) | 0(0) | 38(86,4) | 0(0) | 44(100) | 0(0) | 0(0) |
| **Kaneko^1^ (2014)[63]** | Observational | 114 | 61.6 | 82 (71,9) | 12(10,5) | 4(3,5) | 98(86) | 0(0) | 114(100) | 0(0) | 0(0) |
| **Preventza^1^ (2017) [175]** | Observational | 140 | 53.5 | 111 (79,3) | 0(0) | 0(0) | 140(100) | 0(0) | 140(100) | 0(0) | 0(0) |
| **Misfeld^1^ (2012)[176]** | Observational | 123 | 65 | 82 (66,7) | 51(41,5) | (0) | 68(55,3) | 0(0) | 123(100) | 0(0) | 0(0) |
| **Tarola^1^ (2018) [177]** | Observational | 44 | 66 | 31 (70,5) | 10(22,7) | 1(0) | 27(61,4) | 0(0) | 44(100) | 0(0) | 0(0) |
| **Norton^1^ (2020) [178]** | Observational | 140 | 59.5 | 94 (67,1) | 140(100) | 0(0) | 0(0) | 0(0) | 140(100) | 0(0) | 0(0) |
| **Peterson^1^ (2020) [179]** | Observational | 111 | 631 | 85 (76,6) | 0(0) | 0(0) | 111(100) | 0(0) | 111(100) | 0(0) | 0(0) |
| **Strauch^1^ (2004) [180]** | Observational | 67 | 68 | 30 (44,8) | 8(11,9) | 10(0) | 49(73,1) | 0(0) | 67(0) | 0(0) | 0(0) |
| **Zhang^1^**  **(2020) [181]** | Observational | 258 | 47.9 | 196 (76) | 258(100) | 0(0) | 0(0) | 258(100) | 0(0) | 0(0) | 0(0) |
| **Harrington^2^**  **(2004) [182]** | RCT | 21 | 67 | 12 (57,1) | 0(0) | 3(0) | 16(76,2) | 21(100) | 0(0) | 0(0) | 0(0) |
| **Mourad^2^ (2016) [183]** | Observational | 73 | 69.2 | 43 (58,9) | 0(0) | 0(0) | 73(100) | 73(100) | 0(0) | 0(0) | 0(0) |
| **Preventza^2^ (2015) [184]** | Observational | 137 | 58 | 105 (76,6) | 137(100) | 0(0) | 0(0) | 137(100) | 0(0) | 0(0) | 0(0) |
| **Misfeld^2^ (2012) [176]** | Observational | 242 | 62 | 152 (62,8) | 98(40,5) | 0(0) | 128(52,9) | 242(100) | 0(0) | 0(0) | 0(0) |
| **Norton^2^ (2020) [178]** | Observational | 167 | 57 | 123 (73,7) | 167(100) | 0(0) | 0(0) | 167(100) | 0(0) | 0(0) | 0(0) |
| **Chu^2^**  **(2019) [185]** | Observational | 40 | 66 | 22 (55) | 4(10) | 17(0) | 39(97,5) | 40(100) | 0(0) | 0(0) | 0(0) |
| **Della Corte^2^ (2006) [186]** | Observational | 122 | 59.2 | 82 (67,2) | 102(83,6) | 0(0) | 20(16,4) | 122(100) | 0(0) | 0(0) | 0(0) |
| **Okita^2^**  **(2001) [187]** | Observational | 30 | 67.6 | 25 (83,3) | 0(0) | 5(0) | 25(83,3) | 30(100) | 0(0) | 0(0) | 0(0) |
| **Hata^2^ (2009) [188]** | Observational | 43 | 69.2 | 13 (30,2) | 43(100) | 0(0) | 0(0) | 43(100) | 0(0) | 0(0) | 0(0) |
| **Perreas^3^ (2012) [189]** | Observational | 207 | 63,5 | 162 (78,3) | 102(49,3) | 17(0) | 88(42,5) | 0(0) | 0(0) | 207(100) | 0(0) |
| **Muller^3^ (2004) [190]** | Observational | 22 | 64 | 11 (50) | 11(50) | 0(0) | 1(4,5) | 0(0) | 0(0) | 22(100) | 0(0) |
| **Estrera^3^ (2010) [191]** | Observational | 34 | 56 | 53 (155,9) | 8(23,5) | 9(0) | 0(0) | 0(0) | 0(0) | 34(100) | 0(0) |
| **Yagdi^3^ (2000) [192]** | Observational | 144 | 53.4 | 110 (76,4) | 95(66) | 0(0) | 49(34) | 0(0) | 0(0) | 144(100) | 0(0) |
| **Misfeld^3^ (2012) [176]** | Observational | 51 | 62 | 38 (74,5) | 14(27,5) | 0(0) | 36(70,6) | 0(0) | 0(0) | 51(100) | 0(0) |
| **Moshkovitz^3^ (1998) [193]** | Observational | 104 | 60,5 | 43 (41,3) | 13(12,5) | 14(0) | 69(66,3) | 0(0) | 0(0) | 104(100) | 0(0) |
| **Urbanski^4^ (2003) [194]** | Observational | 43 | 58.9 | 28 (65,1) | 43(100) | 0(0) | 0(0) | 0(0) | 0(0) | 0(0) | 43(100) |
| **Harrington^4^ (2004) [182]** | RCT | 21 | 60 | 14 (66,7) | 1(4,8) | 2(0) | 17(81) | 0(0) | 0(0) | 0(0) | 21(100) |
| **Muller^4^ (2004)[190]** | Observational | 12 | 62 | 5 (41,7) | 8(66,7) | 0(0) | 4(33,3) | 0(0) | 0(0) | 0(0) | 12(100) |
| **Misfeld^4^ (2012)**  **[176]** | Observational | 220 | 62 | 136 (61,8) | 71(32,3) | 0(0) | 143(65) | 0(0) | 0(0) | 0(0) | 220(100) |
| **Ehrlich^4^ (2000) [195]** | Observational | 124 | 59 | 84 (67,7) | 124(100) | 0(0) | 0(0) | 0(0) | 0(0) | 0(0) | 124(100) |
| **Norton^5^**  **(2020) [178]** | Observational | 307 | 58 | 217 (70,7) | 307(100) | 0(0) | 0(0) | 167(3) | 140(0) | 0(0) | 0(0) |
| **Stamou^5^ (2016) [196]** | Observational | 323 | 59 | 223 (69) | 323(100) | 0(0) | 0(0) | 0(0) | 84(0) | 55(17) | 184(57) |
| **Di Eusanio^5^ (2003) [197]** | Observational | 122 | 61.2 | 76 (62,3) | 122(100) | 0(0) | 0(0) | 122(100) | 0(0) | 0(0) | 0(0) |
| **Kondo^5^ (2019) [198]** | Observational | 44 | 83.5 | 12 (27,3) | 44(100) | 0(0) | 0(0) | 44(4) | 0(0) | 0(0) | 0(0) |
| **Inoue^5^ (2016) [199]** | Observational | 334 | 67 | 159 (47,6) | 334(100) | 0(0) | 0(0) | 334(100) | 0(0) | 0(0) | 0(0) |
| **Bavaria^5^ (2002) [200]** | Observational | 163 | 62 | 111 (68,1) | 163(100) | 0(0) | 0(0) | 7(4,3) | 0(0) | 156(95,7) | 0(0) |
| **Vallabhajosyula^5^ (2015) [201]** | Observational | 31 |  | 20 (64,5) | 31(100) | 0(0) | 0(0) | 0(0) | 10(0) | 0(0) | 0(0) |
| **Luo^5^ (2009) [202]** | Observational | 196 | 57 | 142 (72,4) | 196(100) | 0(0) | 0(0) | 0(0) | 168(0) | 0(0) | 0(0) |
| **Algarni^5^ (2014) [203]** | Observational | 128 | 60.6 | 61 (47,7) | 128(100) | 0(0) | 0(0) | 0(0) | 22(0) | 73(57) | 33(25,8) |
| **Saritas^6^ (2010) [204]** | Observational | 50 | 56 | 17 (34) | 0(0) | 37(0) | 13(26) | 0(0) | 50(0) | 0(0) | 0(0) |
| **Leschnower^6^ (2013) [205]** | Observational | 464 | 59.5 | 323 (69,6) | 0(0) | 0(0) | 464(100) | 0(0) | 464(0) | 0(0) | 0(0) |
| **Ma^6^ (2014) [206]** | Observational | 347 | 45.9 | 264 (76,1) | 0(0) | 347(0) | 0(0) | 0(0) | 347(0) | 0(0) | 0(0) |
| **Czerny^6^ (2013) [207]** | Observational | 39 | 63 | 31 (79,5) | 0(0) | 14(0) | 23(59) | 37(7) | 0(0) | 0(0) | 2(5,1) |
| **Di Eusanio^6^ (2013) [208]** | Observational | 57 | 64.8 | 37 (64,9) | 0(0) | 0(0) | 57(100) | 57(100) | 0(0) | 0(0) | 0(0) |
| **Beckmann^6^ (2021) [209]** | Observational | 225 | 64 | 139 (61,8) | 0(0) | 96(0) | 125(55,6) | 225(100) | 0(0) | 0(0) | 0(0) |
| **Okada^6^ (2014) [210]** | Observational | 229 | 71.7 | 175 (76,4) | 0(0) | 24(0) | 205(89,5) | 229(100) | 0(0) | 0(0) | 0(0) |
| **Chu^6^ (2016) [211]** | Observational | 140 | 62.9 | 101 (72,1) | 0(0) | 0(0) | 140(100) | 0(0) | 140(0) | 0(0) | 0(0) |
| **Spielvogel^6^ (2005) [212]** | Observational | 109 | 63.9 | 62 (56,9) | 0(0) | 35(0) | 71(65,1) | 0(0) | 109(0) | 0(0) | 0(0) |
| **Iba^6^ (2014) [213]** | Observational | 143 | 72.1 | 117 (81.8) | 0(0) | 0(0) | 143(100) | 143(100) | 0(0) | 0(0) | 0(0) |
| **Garg^6^ (2014) [214]** | Observational | 50 | 60 | 38 (76) | 0(0) | 0(0) | 50(100) | 0(0) | 50(0) | 0(0) | 0(0) |

^1^ Only unilateral cerebral perfusion subgroup extracted

^2^ Only bilateral cerebral perfusion subgroup extracted

^3^ Only retrograde cerebral perfusion subgroup extracted

^4^ Only deep hypothermic circulatory arrest subgroup extracted

^5^ Only type A aortic dissection subgroup extracted

^6^ Only aneurysm subgroup extracted

**Supplementary Table 3:** Meta regression for mortality in overall group

| **Characteristics** | **Beta (95% CI)** | **N studies** | **P value** | **Explained heterogeneity (%)** |
| --- | --- | --- | --- | --- |
| Age | -0.03 (-.05; -0.008) | 167 | 0.004 | 1.3% |
| Male | 0.42 (-0.64; 1.48) | 163 | 0.44 | 0.0% |
| Acute dissections | 1.13 (0.86; 1.40) | 164 | <0.0001 | 34.70% |
| Chronic dissections | -0.33 (-1.19; 0.54) | 164 | 0.46 | 0.00% |
| Degenerative | -1.24 (-1.54; -0.94) | 165 | <0.0001 | 35.35% |
| Hemiarch replacement | -0.26 (-0.56; 0.04) | 164 | 0.09 | 0.0% |
| Total aortic arch replacement | 0.21 (-0.09; 0.50) | 165 | 0.17 | 0.0% |
| ET | 0.22 (-0.52; 0.96) | 168 | 0.57 | 0.0% |
| FET | 0.12 (-0.25; 0.49) | 168 | 0.52 | 0.0% |
| Hypertension | -0.43 (-1.45; 0.59) | 108 | 0.41 | 0.00% |
| Emergency | 1.04 (0.46; 1.63) | 85 | 0.0004 | 18.33% |
| History of CVA/TIA | -2.26 (-4.27; -0.24) | 97 | 0.0279 | 1.66% |
| Marfan | -0.28 (-2.64; 2.07) | 70 | 0.81 | 0.00% |
| COPD | -0.99 (-2.24;0.27) | 92 | 0.12 | 4.10% |
| Previous cardiac surgery | -0.22 (-1.74; 1.30) | 100 | 0.78 | 0.00% |
| CAD | -2.09 (-3.75; -0.43) | 75 | 0.0138 | 6.07% |
| DM | 1.77 (-0.55; 4.10) | 95 | 0.14 | 0.00% |
| DHCA | 0.21 (-0.18; 0.59) | 162 | 0.29 | 0.00% |
| Unilateral ACP | -0.17 (-0.47; 0.14) | 161 | 0.29 | 0.00% |
| Bilateral ACP | 0.07 (-0.20;0.34) | 163 | 0.60 | 0.00% |
| RCP | -0.16 (-0.58; 0.26) | 162 | 0.45 | 0.00% |
| CPB time | 0.0035 (0.0009; 0.0062) | 160 | 0.0088 | 1.08% |
| ACC time | 0.0033 (-0.0013;0.0078) | 135 | 0.16 | 0.00% |
| Lowest rectal temperature | -0.0601 (-0.0961; -0.0241) | 129 | 0.0011 | 7.62% |
| Year of publication | -0.0283 (-0.0440;-0.0126) | 168 | 0.0004 | 5.61% |

Each meta-regression model included each covariate as the explanatory variable. Β coefficient represents the % change in the outcome risk per unit increase in the relevant variable.

**Supplementary Table 4:** Meta regression for permanent CVA in overall group

| **Characteristics** | **Beta (95% CI)** | **N studies** | **P value** | **Explained heterogeneity (%)** |
| --- | --- | --- | --- | --- |
| Age | -0.0048 (-0.0276; 0.0181) | 143 | 0.68 | 0.0% |
| Male | 0.2157 (-1.1898; 1.6212) | 141 | 0.76 | 0.0% |
| Acute dissections | 0.7882 (0.4154; 1.1611) | 139 | <.0001 | 14.60% |
| Chronic dissections | -0.1509 (-1.1619; 0.8601) | 139 | 0.77 | 0.0% |
| Degenerative | -0.8818 (-1.2866; -0.4770) | 140 | <.0001 | 13.12% |
| Hemiarch replacement | 0.0486 (-0.3181; 0.4153) | 140 | 0.80 | 0.39% |
| Total aortic arch replacement | -0.0794 (-0.4433; 0.2845) | 141 | 0.67 | 1.46% |
| ET | -0.0999 (-1.0409; 0.8411) | 143 | 0.84 | 0.0% |
| FET | 0.1423 (-0.3056; 0.5902) | 143 | 0.53 | 0.0% |
| Hypertension | 0.1470 (-1.0718; 1.3658) | 94 | 0.81 | 0.0% |
| Emergency | 1.1906 (0.4693; 1.9119) | 76 | 0.0012 | 17.40% |
| History of CVA/TIA | -3.3089 (-6.2042;-0.4136) | 85 | 0.0251 | 1.31% |
| Marfan | -0.2637 (-2.4911;1.9637) | 63 | 0.82 | 0.11% |
| COPD | -0.7247 (-2.1413; 0.6919) | 83 | 0.32 | 3.14% |
| Previous cardiac surgery | -0.2965 (-2.0353; 1.4423) | 91 | 0.74 | 0.0% |
| CAD | -0.6866 (-2.6138; 1.2406) | 65 | 0.49 | 0.0% |
| DM | 1.0752 (-1.5573; 3.7078) | 83 | 0.42 | 0.0% |
| DHCA | -0.2698 (-0.7767; 0.2371) | 138 | 0.30 | 2.5% |
| Unilateral ACP | -0.3521 (-0.7204; 0.0162) | 136 | 0.0610 | 2.06% |
| Bilateral ACP | 0.3072 (-0.0139; 0.6283) | 139 | 0.0608 | 1.19% |
| RCP | 0.1222 (-0.4047; 0.6492) | 138 | 0.65 | 0.0% |
| CPB time | 0.0026 (-0.0003; 0.0054) | 137 | 0.0742 | 0.95% |
| ACC time | 0.0034 (-0.0015; 0.0082) | 115 | 0.17 | 0.0% |
| Lowest rectal temperature | -0.0222 (-0.0670; 0.0225) | 115 | 0.33 | 1.26% |
| Year of publication | -0.0053 (-0.0269; 0.0163) | 143 | 0.63 | 0.0% |

Each meta-regression model included each covariate as the explanatory variable. Β coefficient represents the % change in the outcome risk per unit increase in the relevant variable.

**Supplementary Table 5**: Outcomes after Sensitivity analyses in which studies with sample size below 25th quantile are excluded

| **Characteristic** | **Overall** | | **Bilateral ACP** | | **Unilateral ACP** | | **RCP** | | **DHCA** | |
| --- | --- | --- | --- | --- | --- | --- | --- | --- | --- | --- |
|  | Pooled estimate | N studies (I^2^) | Pooled estimate | N studies (I^2^) | Pooled estimate | N studies (I^2^) | Pooled estimate | N studies (I^2^) | Pooled estimate | N studies (I^2^) |
| Operative mortality | 7.8 (7.0-8.8) | 126 (88.9%) | 8.9 (7.5-10.3) | 55 (80.5%) | 6.2(4.9-7.9) | 35 (79.6%) | 7.1 (4.8-10.4) | 20 (84.9%) | 8.4 (5.8-12.0) | 19 (91.6%) |
| Disabeling stroke | 5.8 (5.0-6.7) | 106 (89.3%) | 7.1 (6.0-8.4) | 52 (78.1%) | 4.3 (3.3-5.7) | 33 (74.2%) | 5.6 (3.8-8.2) | 19 (79.8%) | 5.6 (3.6-8.4) | 15 (89.7%) |
| TIA | 7.1 (6.0-8.5) | 77 (91.9%) | 8.1 (6.5-10.0) | 36 (84.6%) | 6.9 (4.9-9.7) | 24 (83.0%) | 6.3 (3.2-11.9) | 12 (90.1%) | 5.5 (2.9-10.1) | 13 (94.7%) |
| Paraplegia | 2.2 (1.6-3.1) | 39 (75.3%) | 2.2 (1.5-3.2) | 26 (53.8%) | 2.1 (1.4-3.4) | 9 (45.2%) | - | 0 (-) | - | - |
| Resternotomy | 7.8 (6.7-8.9) | 79 (86.4%) | 7.2 (5.7-8.9) | 34 (78.7%) | 6.4 (5.0 -8.0) | 27 (77.4%) | 6.7 (4.1-10.8) | 14 (87.9%) | 6.3 (4.7-8.6) | 12 (71.7%) |
| Dialysis | 6.7 (5.2-8.4) | 48 (93.4%) | 6.5 (4.5-9.4) | 21 (88.1%) | 5.9 (3.8-9.1) | 14 (91.5%) | 5.5 (3.2-9.3) | 7 (79.4%) | 4.3 (1.7-10.5) | 4 (94.9%) |
| Respiratory failure | 16.6 (14.3-19.2) | 66 (94.7%) | 20.0 (16.0-24.7) | 36 (94.4%) | 15.0 (9.7-22.5) | 20 (95.4%) | 17.0 (11.4-24.4) | 12 (91.5%) | 12.1 (5.9-23.0) | 7 (94.ˆ%) |
| Tracheostoma | 7.4 (5.5-9.9) | 26 (91.3%) | 7.7 (5.2-11.4) | 12 (77.8%) | 7.7 (3.7-15.4) | 9 (94.3%) | 4.0 (1.3-11.5) | 3 (40.3%) | 3.0 (1.3-6.6) | 4 (91.3%) |
| New onset AF | 20.2 (12.4- 31.2) | 9 (96.3%) | 24.7 (16.7-35.0) | 2 (-) | - | 0 (-) | 35.7 (16.3-61.3) | 2 (-) | - | - |
| AKI | 10.1 (8.1-12.4) | 48 (92.9%) | 13.2 (1.0-18.8) | 21 (93.2%) | 9.6 (7.1-12.8) | 18 (70.9%) | 11.0 (0.7-16.8) | 13 (92.3%) | 6.5 (3.3-12.6) | 4 (83.7%) |
| Mediastinitis | 2.6 (2.0- 3.4) | 36 (74.8%) | 3.5 (2.4-5.1) | 18 (71.2%) | 4.7 (3.1-7.2) | 7 (8.5%) | 1.1 (0.2-6.3) | 4 (76.5%) | 1.6 (1.1-2.3) | 3 (0%) |
| Pacemaker | 7.6 (2.3- 22.2) | 6 (96.5%) | 3.7 (2.2-6.1) | 2 (-) | 3.2 (1.0-10.2) | 4 (75.0%) | - | - | - | - |
| ICU stay (days) | 6.3 (5.6 -7.0) | 34 (98.2%) | 7.4 (6.0-8.9) | 14 (96.2%) | 5.4 (4.7-6.1) | 14 (99.6%) | 4.1 (2.0-6.1) | 2 (-) | 8.2 (2.7-13.8) | 3 (96.4%) |
| Hospital stay (days) | 19.9 (17.4-22.3) | 29 (99.0%) | 22.6 (18.8-26.4) | 13 (98.1%) | 18.5 (13.9-23.1) | 10 (98.8%) | 11.2 (8.2-14.1) | 5 (88.8%) | 13.5 (10.3-16.8) | 6 (98.6%) |

AF, atrial fibrillation; AKI, acute kidney insufficiency; ICU, intensive care unit; TIA, transient ischemic attack.

**Supplementary Table 6**: Outcome of aneurysm and acute dissection subgroups

| **Characteristic** | **Aneurysms** | | **Acute dissections** | |
| --- | --- | --- | --- | --- |
|  | Pooled estimate | N studies (I^2^) | Pooled estimate | N studies (I^2^) |
| Operative mortality | 4.5 (3.6-5.5) | 45 (72.2%) | 12.7 (10.6-15.3) | 41 (80.0%) |
| CVA permanent | 3.8 (2.8-5.3) | 35 (83.9%) | 7.9 (6.3-9.9) | 35 (73.8%) |
| CVA transient | 7.3 (5.5-9.7) | 22 (82.7%) | 13.6 (9.8-18.5) | 18 (82.7%) |
| Paraplegia | 2.7 (1.8-3.8) | 14 (38.4%) | 1.9 (0.9-4.0) | 9 (41.9%) |
| Restornotomy | 6.5 (5.1-8.4) | 28 (80.4%) | 10.9 (8.7-13.6) | 28 (74.3%) |
| Dialysis | 4.9 (3.1-7.7) | 18 (89.5%) | 13.0 (8.5-19.4) | 13 (91.2%) |
| Respiratory failure | 13.2 (10.3-16.8) | 29 (91.1%) | 22.6 (16.6-30.0) | 23 (94.4%) |
| Tracheostoma | 7.2 (4.3-11.9) | 7 (91.7%) | 8.0 (5.0-12.6) | 11 (88.2%) |
| New onset AF | 26.0 (18.9-34.8) | 2 (37.6%) | 21.5 (18.0-25.4) | 3 (0.0%) |
| AKI | 9.2 (6.1-13.6) | 21 (92.3%) | 16.8 (10.8-25.2) | 12 (86.2%) |
| Mediastinitis | 3.5 (1.9 -6.1) | 9 (67.8%) | 3.8 (2.3-6.0) | 12 (63.8%) |
| Pacemaker | 4.9 (1.1-18.5) | 2 (41.1%) | 5.7 (3.9-8.3) | 2 (0.0%) |
| ICU stay | 3.8 (2.4-5.3) | 7 (91.8%) | 6.9 (5.9-8.0) | 15 (97.2%) |
| Hospital stay | 17.1 (13.6-20.6) | 10 (98.4%) | 19.4 (17.0-21.9) | 15 (96.5%) |

Each meta-regression model included each covariate as the explanatory variable. Β coefficient represents the % change in the outcome risk per unit increase in the relevant variable.

**Supplementary Table 7:** Meta regression for operative mortality in aneurysms

| **Characteristics** | **Beta (95% CI)** | **N studies** | **P value** | **Explained heterogeneity (%)** |
| --- | --- | --- | --- | --- |
| Age | -0.0032 (-0.037; 0.031) | 45 | 0.85 | 0.0% |
| Male | -0.66 (-2.46; 1.14) | 44 | 0.47 | 0.2% |
| Hemiarch replacement | -0.9077 (-1.4144; -0.4010) | 45 | 0.0004 | 20.72% |
| Total aortic arch replacement | 0.9077 (0.4010; 1.4144) | 45 | 0.0004 | 20.72% |
| ET | 0.4197 (-0.5331; 1.3725) | 44 | 0.3879 | 0.0% |
| FET | 0.7776 (0.1349; 1.4204) | 44 | 0.0177 | 25.61% |
| Hypertension | 0.17 (-1.51; 1.84) | 31 | 0.84 | 0.0% |
| History of CVA/TIA | 1.52 (-1.007; 4.05) | 29 | 0.24 | 0.0% |
| Marfan | 2.36 (-0.59; 5.32) | 14 | 0.12 | 2.7% |
| COPD | 1.68 (-0.98; 4.33) | 31 | 0.22 | 1.9% |
| Previous cardiac surgery | 1.58 (-0.34; 3.50) | 29 | 0.11 | 22.3% |
| CAD | 0.040 (-2.83; 2.91) | 19 | 0.98 | 0.0% |
| DM | -1.71 (-6.44; 3.01) | 33 | 0.48 | 0.0% |
| DHCA | -0.30 (-1.16; 0.56) | 45 | 0.50 | 0.0% |
| Unilateral ACP | -0.36 (-0.91; 0.19) | 45 | 0.20 | 0.0% |
| Bilateral ACP | 0.67 (0.26; 1.09) | 45 | 0.0016 | 33.0% |
| RCP | -0.90 (-1.65; -0.16) | 45 | 0.017 | 15.2% |
| CPB time | 0.0045 (-0.0014; 0.010) | 42 | 0.13 | 0.0% |
| ACC time | -0.0003 (-0.011; 0.010) | 38 | 0.95 | 0.0% |
| Lowest rectal temperature | -0.0053 (-0.095; 0.085) | 30 | 0.91 | 0.0% |
| Year of publication | -0.014 (-0.049; 0.021) | 45 | 0.44 | 0.0% |

Each meta-regression model included each covariate as the explanatory variable. Β coefficient represents the % change in the outcome risk per unit increase in the relevant variable.

**Supplementary Table 8:** Meta regression for disabling stroke in aneurysms

| **Characteristics** | **Beta (95% CI)** | **N studies** | **P value** | **Explained heterogeneity (%)** |
| --- | --- | --- | --- | --- |
| Age | 0.0133 (-0.0403; 0.0669) | 35 | 0.63 | 0.0% |
| Male | -1.6171 (-4.7030; 1.4688) | 35 | 0.30 | 14.59% |
| Hemiarch replacement | -0.6903 (-1.4506; 0.0700) | 35 | 0.0751 | 1.12% |
| Total aortic arch replacement | 0.6903 (-0.0700; 1.4506) | 35 | 0.0751 | 1.12% |
| ET | 0.5256 (-0.7650; 1.8161) | 34 | 0.4248 | 0.0% |
| FET | 0.7341 (-0.2039; 1.6722) | 34 | 0.1250 |  |
| Hypertension | -0.0158 (-2.8420; 2.8105) | 25 | 0.9913 | 8.04% |
| Emergency | 3.3198 (-6.9965; 13.6361) | 8 | 0.53 | 0.0% |
| History of CVA/TIA | 0.5284 (-4.6701; 5.7269) | 24 | 0.84 | 0.0% |
| Marfan | 1.4813 (-2.0830; 5.0456) | 12 | 0.42 | 0.0% |
| COPD | -0.4694 (-6.9612; 6.0225) | 24 | 0.89 | 0.0% |
| Previous cardiac surgery | 1.6500 (-1.1767; 4.4767) | 24 | 0.25 | 0.0% |
| CAD | 3.5785 (-0.6795; 7.8365) | 15 | 0.0995 | 0.0% |
| DM | -1.4427 (-8.9965; 6.1112) | 26 | 0.71 | 0.0% |
| DHCA | 1.1510 (0.4632; 2.7652) | 35 | 0.16 | 0.0% |
| Unilateral ACP | -0.8269 (-1.5056; -0.1482) | 35 | 0.0169 | 29.03% |
| Bilateral ACP | 0.6855 (0.0933; 1.2778) | 35 | 0.0233 | 33.62% |
| RCP | -0.5723 (-1.5529; 0.4083) | 35 | 0.25 | 14.79% |
| CPB time | 0.0050 (-0.0041; 0.0141) | 33 | 0.28 | 0.0% |
| ACC time | 0.0017 (-0.0128; 0.0163) | 29 | 0.82 | 0.0% |
| Lowest rectal temperature | -0.0412 (-0.1650; 0.0825) | 26 | 0.51 | 0.0% |
| Year of publication | 0.0229 (-0.0333; 0.0792) | 35 | 0.42 | 10.3% |

Each meta-regression model included each covariate as the explanatory variable. Β coefficient represents the % change in the outcome risk per unit increase in the relevant variable.

**Supplementary Table 9:** Meta regression for operative mortality in acute dissections

| **Characteristics** | **Beta (95% CI)** | **N studies** | **P value** | **Explained heterogeneity (%)** |
| --- | --- | --- | --- | --- |
| Age | 0.012 (-0.013; 0.037) | 40 | 0.33 | 0.6% |
| Male | 0.27 (-1.35; 1.90) | 39 | 0.74 | 0.0% |
| Hemiarch replacement | -0.9077 (-1.4144; -0.4010) | 45 | 0.0004 | 20.72% |
| Total aortic arc replacement | 0.9077 (0.4010; 1.4144) | 45 | 0.0004 | 20.72% |
| ET | 0.4197 (-0.5331; 1.3725) | 44 | 0.39 | 0.0% |
| FET | 0.7776 (0.1349; 1.4204) | 44 | 0.0177 | 25.61% |
| Hypertension | 0.35 (-1.53; 2.22) | 26 | 0.72 | 0.0% |
| Emergency | -1.31 (-3.61; 1.00) | 6 | 0.27 | 0.0% |
| History of CVA/TIA | -2.24 (-9.69; 5.22) | 19 | 0.56 | 0.0% |
| Marfan | -1.24 (-10.71; 8.22) | 22 | 0.80 | 0.0% |
| COPD | -0.27 (-3.22; 2.68) | 20 | 0.86 | 0.0% |
| Previous cardiac surgery | 1.71 (-5.66; 9.08) | 16 | 0.65 | 0.0% |
| CAD | 2.30 (-1.51; 6.11) | 17 | 0.24 | 15.3% |
| DM | 2.55 (-2.03; 7.14) | 24 | 0.27 | 1.2% |
| DHCA | 0.28 (-0.39; 0.95) | 41 | 0.41 | 0.0% |
| Unilateral ACP | 0.21 (-0.41; 0.82) | 41 | 0.51 | 0.0% |
| Bilateral ACP | -0.37 (-0.85; 0.11) | 41 | 0.13 | 0.5% |
| RCP | 0.11 (-0.77; 0.98) | 41 | 0.81 | 0.0% |
| CPB time | 0.006 (0.005; 0.012) | 39 | 0.033 | 0.0% |
| ACC time | 0.0031 (-0.007; 0.013) | 31 | 0.54 | 0.0% |
| Lowest rectal temperature | -0.0998 (-0.18; -0.019) | 29 | 0.015 | 1.7% |
| Year of publication | -0.027 (-0.055; 0.0013) | 41 | 0.062 | 2.6% |

Each meta-regression model included each covariate as the explanatory variable. Β coefficient represents the % change in the outcome risk per unit increase in the relevant variable.

**Supplementary Table 10**: Meta regression for disabling stroke in acute dissections

| **Characteristics** | **Beta (95% CI)** | **N studies** | **P value** | **Explained heterogeneity (%)** |
| --- | --- | --- | --- | --- |
| Age | 0.0233 (-0.0042; 0.0509) | 34 | 0.0966 | 10.13% |
| Male | -0.3565 (-2.2016; 1.4886) | 35 | 0.70 | 0.0% |
| Hemiarch replacement | -0.6903 (-1.4506; 0.0700) | 35 | 0.0751 | 1.12% |
| Total aortic arch replacement | 0.6903 (-0.0700; 1.4506) | 35 | 0.0751 | 1.12% |
| ET | 0.5256 (-0.7650; 1.8161) | 34 | 0.42 | 0.0% |
| FET | 0.7341 (-0.2039; 1.6722) | 34 | 0.13 | 8.55% |
| Hypertension | 0.9490 (-1.1932; 3.0911) | 23 | 0.39 | 0.0% |
| Emergency | 0.9356 (-1.1361; 3.0074) | 6 | 0.38 | 0.0% |
| History of CVA/TIA | -7.0185 (-13.8847; -0.1523) | 15 | 0.0451 | 39.70% |
| Marfan | -3.4791 (-10.7121; 3.7539) | 19 | 0.35 | 13.45% |
| COPD | 3.2511 (0.4641; 6.0381) | 16 | 0.0222 | 21.20% |
| Previous cardiac surgery | 0.1753 (-6.5600; 6.9107) | 16 | 0.9593 | 0.0% |
| CAD | -0.6866 (-5.7321; 4.3589) | 15 | 0.79 | 0.0% |
| DM | 1.5084 (-3.5143; 6.5310) | 21 | 0.56 | 1.84% |
| DHCA | 0.0384 (-0.9567; 1.0335) | 35 | 0.94 | 0.0% |
| Unilateral ACP | -0.1916 (-0.8561; 0.4728) | 35 | 0.57 | 0.0% |
| Bilateral ACP | 0.0572 (-0.5076; 0.6220) | 35 | 0.84 | 0.0% |
| RCP | 0.2791 (-0.7695; 1.3277) | 35 | 0.60 | 0.0% |
| CPB time | 0.0050 (-0.0013; 0.0113) | 33 | 0.12 | 0.0% |
| ACC time | 0.0037 (-0.0078; 0.0152) | 26 | 0.53 | 0.0% |
| Lowest rectal temperature | -0.0774 (-0.1792; 0.0244) | 24 | 0.14 | 0.0% |
| Year of publication | -0.0121 (-0.0457; 0.0214) | 35 | 0.48 | 0.0% |

Each meta-regression model included each covariate as the explanatory variable. Β coefficient represents the % change in the outcome risk per unit increase in the relevant variable.

**Supplementary text 1: Search terms**

| embase.com | 2410 | 2382 |
| --- | --- | --- |
| Medline Ovid | 1820 | 218 |
| Web of science | 1453 | 266 |
| Cochrane CENTRAL | 68 | 28 |
| Google scholar | 200 | 53 |
| **Total** | **5951** | **2947** |

**Embase.com**

('aortic arch surgery'/de OR 'aortic arch anomaly'/exp/dm_su OR 'aortic trauma'/exp/dm_su OR (('aortic disease'/exp/dm_su OR 'aortic surgery'/exp OR 'aortic reconstruction'/exp OR 'aortic trauma'/exp OR 'aortic arch anomaly'/exp) AND ('thoracic aorta'/de OR 'aortic arch'/de OR 'ascending aorta'/de)) OR (((arch* OR hemiarch*) NEAR/10 (surg* OR reconstruct* OR repair* OR replace* OR graft* OR operat* OR reoperat*)) OR (Elephant* NEAR/3 Trunk*)):ab,ti) AND ('deep hypothermic circulatory arrest'/exp OR 'moderate hypothermic circulatory arrest'/de OR 'mild hypothermic circulatory arrest'/de OR (('heart arrest'/de OR 'brain perfusion'/de) AND ('hypothermia'/de OR 'induced hypothermia'/de)) OR (((circulator*) NEAR/3 arrest*) OR ((hypotherm*) NEAR/6 (heart OR cardiac) NEAR/6 (arrest)) OR ((hypotherm*) NEAR/6 (brain OR cerebral OR antegrade OR retrograde) NEAR/6 (perfusion)) OR ((deep OR moderate OR mild) NEAR/3 hypotherm*) OR aort*-arch-replace*):ab,ti) NOT (juvenile/exp NOT adult/exp) NOT ([animals]/lim NOT [humans]/lim) NOT ([Conference Abstract]/lim AND [1800-2015]/py) AND [English]/lim

**Medline Ovid**

(exp Aortic Arch Syndromes/su OR ((exp Aortic Diseases/su) AND (Aorta, Thoracic/)) OR (((arch* OR hemiarch*) ADJ10 (surg* OR reconstruct* OR repair* OR replace* OR graft* OR operat* OR reoperat*)) OR (Elephant* ADJ3 Trunk*)).ab,ti.) AND (Circulatory Arrest, Deep Hypothermia Induced/ OR ((Brain/) AND (Perfusion/) AND (Hypothermia/ OR Hypothermia, Induced/)) OR (((circulator*) ADJ3 arrest*) OR ((hypotherm*) ADJ6 (heart OR cardiac) ADJ6 (arrest)) OR ((hypotherm*) ADJ6 (brain OR cerebral OR antegrade OR retrograde) ADJ6 (perfusion)) OR ((deep OR moderate OR mild) ADJ3 hypotherm*) OR aort*-arch-replace*).ab,ti.) NOT ((exp child/ OR exp infant/ OR adolescent/) NOT exp adult/) NOT (exp animals/ NOT humans/) AND english.la.

**Web of science**

TS=(((((arch* OR hemiarch*) NEAR/10 (surg* OR reconstruct* OR repair* OR replace* OR graft* OR operat* OR reoperat*)) OR (Elephant* NEAR/2 Trunk*))) AND ((((circulator*) NEAR/2 arrest*) OR ((hypotherm*) NEAR/5 (heart OR cardiac) NEAR/5 (arrest)) OR ((hypotherm*) NEAR/5 (brain OR cerebral OR antegrade OR retrograde) NEAR/5 (perfusion)) OR ((deep OR moderate OR mild) NEAR/2 hypotherm*) OR aort*-arch-replace*)) NOT ((animal* OR rat OR rats OR mouse OR mice OR murine OR dog OR dogs OR canine OR cat OR cats OR feline OR rabbit OR cow OR cows OR bovine OR rodent* OR sheep OR ovine OR pig OR swine OR porcine OR veterinar* OR chick* OR zebrafish* OR baboon* OR nonhuman* OR primate* OR cattle* OR goose OR geese OR duck OR macaque* OR avian* OR bird* OR fish*) NOT (human* OR patient* OR women OR woman OR men OR man)) NOT ((child* OR infant* OR adolescen*) NOT (adult*))) AND DT=(article) AND LA=(english)

**Cochrane CENTRAL**

((((arch* OR hemiarch*) NEAR/10 (surg* OR reconstruct* OR repair* OR replace* OR graft* OR operat* OR reoperat*)) OR (Elephant* NEAR/3 Trunk*)):ab,ti) AND ((((circulator*) NEAR/3 arrest*) OR ((hypotherm*) NEAR/6 (heart OR cardiac) NEAR/6 (arrest)) OR ((hypotherm*) NEAR/6 (brain OR cerebral OR antegrade OR retrograde) NEAR/6 (perfusion)) OR ((deep OR moderate OR mild) NEAR/3 hypotherm*) OR aort* NEXT arch NEXT replace*):ab,ti)

**Google scholar**

"arch reconstruction|repair|replacement|operation|surgery" "deep|moderate|mild hypothermic| hypothermia" arrest

**Supplementary References**

[1] Ito H, Mizumoto T, Sawada Y, Fujinaga K, Tempaku H, Yamamoto Y *et al.* *Neuroprotective effect of pressure-oriented flow regulation and pH-stat management in selective antegrade brain perfusion during total aortic arch repair*. Interact Cardiovasc Thorac Surg 2017;**25**:565-70.

[2] Abdelgawad A, Arafat H. *Moderate versus deep hypothermic circulatory arrest for ascending aorta and aortic arch surgeries using open distal anastomosis technique*. J Egypt Soc Cardio-Thorac Surg 2017;**25**:323-30.

[3] Abjigitova D, Mokhles MM, Papageorgiou G, Bekkers JA, Bogers AJJC. *Outcomes of different aortic arch replacement techniques*. J Card Surg 2020;**35**:367-74.

[4] Akpinar B, Güden M, Aytekin S, Sanisoglu I, Sagbas E, Özbek U *et al.* *The use of stentless valves for root replacement during repair of ascending aortic aneurysms with aortic valve regurgitation*. Heart Surg Forum 2002;**5**:52-55.

[5] Alamanni F, Agrifoglio M, Pompilio G, Spirito R, Sala A, Arena V *et al.* *Aortic arch surgery: Pros and cons of selective cerebral perfusion. A multivariable analysis for cerebral injury during hypothermic circulatory arrest*. J cardiovasc surg 1995;**36**:31-37.

[6] Al-Sabri S, Brauer A, Hinz J, Grossman M, Schondube F, Danner CB *et al.* *Abdominal organ dysfunction after open surgical aortic arch replacement using different cerebral protection strategies*. Thorac Cardiovasc Surg 2019;**67**.

[7] Amano K, Takami Y, Ishikawa H, Ishida M, Tochii M, Akita K *et al.* *Lower body ischaemic time is a risk factor for acute kidney injury after surgery for type A acute aortic dissection*. Interact Cardiovasc Thorac Surg 2020;**30**:107-12.

[8] Ando M, Nakajima N, Adachi S, Nakaya M, Kawashima Y. *Simultaneous Graft Replacement of the Ascending Aorta and Total Aortic-Arch for Type-a Aortic Dissection*. Annals of Thoracic Surgery;**57**:669-76.

[9] Apaydin AZ, Islamoglu F, Askar FZ, Engin C, Posacioglu H, Yagdi T *et al.* *Immediate clinical outcome after prolonged periods of brain protection: Retrospective comparison of hypothermic circulatory arrest, retrograde, and antegrade perfusion*. J Card Surg 2009;**24**:486-89.

[10] Apostolakis E, Koletsis EN, Dedeilias P, Kokotsakis JN, Sakellaropoulos G, Psevdi A *et al.* *Antegrade versus retrograde cerebral perfusion in relation to postoperative complications following aortic arch surgery for acute aortic dissection type A*. J Card Surg 2008;**23**:480-87.

[11] Arnaoutakis GJ, Vallabhajosyula P, Bavaria JE, Sultan I, Siki M, Naidu S *et al.* *The Impact of Deep Versus Moderate Hypothermia on Postoperative Kidney Function After Elective Aortic Hemiarch Repair*. Ann Thorac Surg 2016;**102**:1313-21.

[12] Aytekin B, Unal EU, Demir A, Aksu U, Caliskan A, Vardar K *et al.* *Unilateral Antegrade Cerebral Perfusion and Moderate Hypothermia: assessing Safety With Novel Biomarkers*. Heart lung and circulation (no pagination), 2016 2016;**Date of Publication: January 06**.

[13] Bachet J, Guilmet D, Goudot B, Dreyfus GD, Delentdecker P, Brodaty D *et al.* *Antegrade cerebral perfusion with cold blood: A 13-year experience*. Ann Thorac Surg 1999;**67**:1874-78.

[14] Bachet J, Guilmet D. *Brain protection during surgery of the aortic arch*. J Card Surg 2002;**17**:115-24.

[15] Banerjee P, Theus C, Bremerich J, Wolff T, Reuthebuch O, Eckstein F *et al.* *Computed Tomography Scan Predicts Abdominal Interventions but Not Stroke after Surgery for Acute Type A Aortic Dissection*. Thorac Cardiovasc Surg 2015;**64**:108-15.

[16] Bashir M, Field M, Shaw M, Fok M, Harrington D, Kuduvalli M *et al.* *Influences on Early and Medium-Term Survival Following Surgical Repair of the Aortic Arch*. Aorta (Stamford) 2014;**2**:56-73.

[17] Beckmann E, Martens A, Korte W, Kaufeld T, Krueger H, Haverich A *et al.* *Open total arch replacement with trifurcated graft and frozen elephant trunk*. Ann cardiothorac surg 2020;**9**:170-77.

[18] Bjurbom M, Franco-Cereceda A, Liska J, Olsson C. *Outcomes of aortic arch repair with extended (≥ 90 minutes) antegrade cerebral perfusion*. Scand Cardiovasc J 2015;**49**:109-13.

[19] Brat R, Gaj J, Barta J. *Early and mid-term outcomes of the aortic arch surgery: experience from the low-volume centre*. J Cardiothorac Surg 2015;**10**:31.

[20] Casthely PA, Fymann PN, Abrams LM. *Aenesthesia for aortic arch aneurysm repair: Experience with 17 patients*. Can anaesth soc j 1985;**32**:73-78.

[21] Cefarelli M, Murana G, Surace GG, Castrovinci S, Jafrancesco G, Kelder JC *et al.* *Elective Aortic Arch Repair: Factors Influencing Neurologic Outcome in 791 Patients*. Ann Thorac Surg 2017;**104**:2016-23.

[22] Centofanti P, Barbero C, D'Agata F, Caglio MM, Caroppo P, Cicerale A *et al.* *Neurologic and cognitive outcomes after aortic arch operation with hypothermic circulatory arrest*. Surgery 2016;**160**:796-804.

[23] Chen X, Huang F, Xu M, Wang L, Jiang Y, Xiao L *et al.* *The stented elephant trunk procedure combined total arch replacement for Debakey I aortic dissection: Operative result and follow-up*. Interact Cardiovasc Thorac Surg 2010;**11**:594-98.

[24] Chen WS, Ni BQ, Li SQ, Shao YF, Zhang WH. *Novel risk factors for the healthcare associated infections (HAIs) in patients with Stanford type A aortic dissection (TAAD)*. J Thorac Dis 2018;**10**:2135-41.

[25] Chen IM, Chen PL, Weng SH, Hsu CP, Shih CC, Chang HH *et al.* *Clinical Outcomes of VasoRing Connector in Patients With Acute Type A Aortic Dissection*. Ann Thorac Surg 2018;**106**:764-70.

[26] Cheng G, Yan Z, Lu Z, Li J, Wu Y, Lei H *et al.* *Optimal hypothermia conditions for cerebral perfusion in the surgical repair of acute aortic dissection: A single-center pilot clinical study*. Int J Clin Exp Med 2018;**11**:5793-801.

[27] Cho SH, Sung K, Park KH, Yang JH, Kim WS, Jun TG *et al.* *Midterm results of aortic arch replacement in a Stanford type A aortic dissection with an intimal tear in the aortic arch*. Korean Circ J 2009;**39**:270-74.

[28] Chung J, Stevens LM, Ouzounian M, El-Hamamsy I, Bouhout I, Dagenais F *et al.* *Sex-Related Differences in Patients Undergoing Thoracic Aortic Surgery: Evidence from the Canadian Thoracic Aortic Collaborative*. Circulation 2019;**139**:1177-84.

[29] Colli A, Carrozzini M, Galuppo M, Comisso M, Toto F, Gregori D *et al.* *Analysis of early and long-term outcomes of acute type A aortic dissection according to the new international aortic arch surgery study group recommendations*. Heart Vessels 2016;**31**:1616-24.

[30] Cook RC, Gao M, Macnab AJ, Fedoruk LM, Day N, Janusz MT. *Aortic arch reconstruction: Safety of moderate hypothermia and antegrade cerebral perfusion during systemic circulatory arrest*. J Card Surg 2006;**21**:158-64.

[31] Dai XF, Chen LW, Wu XJ, Dong Y, Wang QM. *Total Aortic Arch Reconstruction with Triple-Branched Stent Graft or Hemiarch Replacement for Acute Debakey Type i Aortic Dissection: Five-Years Experience with 93 Patients*. J Card Surg 2015;**30**:749-55.

[32] Damberg A, Carino D, Charilaou P, Peterss S, Tranquilli M, Ziganshin BA *et al.* *Favorable late survival after aortic surgery under straight deep hypothermic circulatory arrest*. J Thorac Cardiovasc Surg 2017;**154**:1831-39.e1.

[33] Danner BC, Natour E, Horst M, Dikov V, Ghosh PK, Dapunt OE. *Comparison of operative techniques in acute type a aortic dissection performing the distal anastomosis*. J Card Surg 2007;**22**:105-10.

[34] Davies RA, Black D, Jeremy RW, Bannon PG, Bayfield MS, Hendel PN *et al.* *Evolution in the Techniques and Outcomes of Aortic Arch Surgery: A 22 Year Single Centre Experience*. Heart Lung Circul 2011;**20**:704-11.

[35] Detter C, Demal TJ, Bax L, Tsilimparis N, Koelbel T, von Kodolitsch Y *et al.* *Simplified frozen elephant trunk technique for combined open and endovascular treatment of extensive aortic diseases*. Eur J Cardiothorac Surg 2019.

[36] Deville C, Roques X, Fern, ez G, Laborde N, Baudet E *et al.* *Should circulatory arrest with deep hypothermia be revised in aortic arch surgery?* Eur J Cardiothorac Surg 1988;**2**:185-91.

[37] Di Eusanio M, Ciano M, Labriola G, Lionetti G, Di Eusanio G. *Cannulation of the innominate artery during surgery of the thoracic aorta: our experience in 55 patients*. European Journal of Cardio-Thoracic Surgery;**32**:270-73.

[38] Di Eusanio M, Schepens M, Morshuis WJ, Di Bartolomeo R, Pierangeli A, Dossche KM. *Antegrade selective cerebral perfusion during operations on the thoracic aorta: Factors influencing survival and neurologic outcome in 413 patients*. Journal of Thoracic and Cardiovascular Surgery 2002;**124**:1080-86.

[39] Dong SB, Xiong JX, Zhang K, Zheng J, Xu SD, Liu YM *et al.* *Different hypothermic and cerebral perfusion strategies in extended arch replacement for acute type a aortic dissection: a retrospective comparative study*. J Cardiothorac Surg 2020;**15**:236.

[40] Ehrlich M, Grabenwöger M, Luckner D, Cartes-Zumelzu F, Simon P, Laufer G *et al.* *The use of profound hypothermia and circulatory arrest in operations On the thoracic aorta*. Eur j cardio-thorac surg 1997;**11**:176-81.

[41] Eldeiry M, Ghincea C, Aftab M, Clevel, C. J, Fullerton D *et al.* *Innominate versus axillary artery cannulation for the hemiarch repair*. J Surg Res 2018;**232**:234-39.

[42] Estrera AL, Miller 3rd CC, Lee TY, Shah P, Safi HJ. *Ascending and transverse aortic arch repair: the impact of retrograde cerebral perfusion*. Circulation 2008;**118**:S160-66.

[43] Farhat F, Durand M, Boussel L, Sanchez I, Villard J, Jegaden O. *Should a reimplantation valve sparing procedure be done systematically in type A aortic dissection?* Eur J Cardio-thorac Surg 2007;**31**:36-41.

[44] Ganapathi AM, Hanna JM, Schechter MA, Englum BR, Castleberry AW, Gaca JG *et al.* *Antegrade versus retrograde cerebral perfusion for hemiarch replacement with deep hypothermic circulatory arrest: Does it matter? A propensity-matched analysis*. J Thorac Cardiovasc Surg 2014;**148**:2896-902.

[45] Gatti G, Benussi B, Curro P, Forti G, Rauber E, Minati A *et al.* *The Risk of Neurological Dysfunctions after Deep Hypothermic Circulatory Arrest with Retrograde Cerebral Perfusion*. Journal of Stroke & Cerebrovascular Diseases 2017;**26**:3009-19.

[46] Ghincea CV, Aftab M, Eldeiry M, Roda GF, Bronsert MR, Pal JD *et al.* *Cardiopulmonary Bypass Time Better Predicts Postoperative Morbidity and Mortality in Aortic Arch Surgery Than Circulatory Arrest Time*. J Am Coll Surg 2019;**229**:e84.

[47] Guo J, Wang Y, Zhu J, Cao J, Chen Z, Li Z *et al.* *Right axillary and femoral artery perfusion with mild hypothermia for aortic arch replacement*. J Cardiothorac Surg 2014;**9**:94.

[48] Hata M, Orime Y, Wakui S, Umeda T, Akiyama K, Tanaka M. *Efficacy of modified less invasive quick replacement using mild hypothermic arrest and partial retrograde cerebral perfusion for type A acute aortic dissection*. Gen Thorac Cardiovasc Surg 2018;**66**:33-37.

[49] Hirano K, Tokui T, Nakamura B, Inoue R, Inagaki M, Hirano R *et al.* *Impact of the Frozen Elephant Trunk Technique on Total Aortic Arch Replacement*. Ann Vasc Surg 2020;**65**:206-16.

[50] Hiraoka A, Chikazawa G, Totsugawa T, Tamura K, Sakaguchi T, Nakajima K *et al.* *Efficacy of right axillary artery perfusion for antegrade cerebral perfusion in open total arch repair*. J Vasc Surg 2014;**60**:436-42.

[51] Ho JYK, Chow SCY, Kwok MWT, Fujikawa T, Wong RHL. *Total Aortic Arch Replacement and Frozen Elephant Trunk*. Semin Thorac Cardiovasc Surg 2020.

[52] Iafrancesco M, Ranasinghe AM, Dronavalli V, Adam DJ, Claridge MW, Riley P *et al.* *Open aortic arch replacement in high-risk patients: The gold standard*. Eur J Cardio-thorac Surg 2016;**49**:646-51.

[53] Iba Y, Minatoya K, Matsuda H, Sasaki H, Tanaka H, Kobayashi J *et al.* *Contemporary open aortic arch repair with selective cerebral perfusion in the era of endovascular aortic repair*. J Thorac Cardiovasc Surg 2013;**145**:S72-S77.

[54] Imasaka KI, Tayama E, Tomita Y. *The impact of carotid or intracranial atherosclerosis on perioperative stroke in patients undergoing open aortic arch surgery*. J Thorac Cardiovasc Surg 2017;**153**:1045-53.

[55] Immer FF, Moser B, Krähenbühl ES, Englberger L, Stalder M, Eckstein FS *et al.* *Arterial Access Through the Right Subclavian Artery in Surgery of the Aortic Arch Improves Neurologic Outcome and Mid-Term Quality of Life*. Ann Thorac Surg 2008;**85**:1614-18.

[56] Inamura S, Furuya H, Yagi K, Ikeya E, Yamaguchi M, Fujimura T *et al.* *Recent surgical outcomes of acute type-A aortic dissection*. Tokai J Exp Clin Med 2006;**31**:109-12.

[57] Jabagi H, Juanda N, Nantsios A, Boodhwani M. *Aortic arch surgery at 32°C: mild hypothermia and unilateral antegrade cerebral perfusion*. Interact Cardiovasc Thorac Surg 2021.

[58] Jacobs MJ, De Mol BA, Veldman DJ. *Aortic arch and proximal supraaortic arterial repair under continuous antegrade cerebral perfusion and moderate hypothermia*. Cardiovasc Surg 2001;**9**:396-402.

[59] Kaku Y, Nakajima M, Ichihara Y, Iizuka K, Tsuchiya K. *Modified elephant trunk technique in distal anastomosis with the aid of antegrade selective cerebral perfusion for total arch replacement*. Ann Thorac Surg 2014;**97**:1281-85.

[60] Kamenskaya OV, Klinkova AS, Chernyavsky AM, Lomivorotov VV, Meshkov IO, Karaskov AM. *Deep hypothermic circulatory arrest vs. Antegrade cerebral perfusion in cerebral protection during the surgical treatment of chronic dissection of the ascending and arch aorta*. J Extra-Corpor Technol 2017;**49**:16-25.

[61] Kan CB, Lin YF, Che PN. *Acute type A aortic dissection repair in a community hospital*. Acta Cardiol Sin 2006;**22**:198-204.

[62] Kaneda T, Saga T, Onoe M, Kitayama H, Nakamoto S, Matsumoto T *et al.* *Antegrade selective cerebral perfusion with mild hypothermic systemic circulatory arrest during thoracic aortic surgery*. Scand Cardiovasc J 2005;**39**:87-90.

[63] Kaneko T, Aranki SF, Neely RC, Yazdchi F, McGurk S, Leacche M *et al.* *Is there a need for adjunct cerebral protection in conjunction with deep hypothermic circulatory arrest during noncomplex hemiarch surgery?* J Thorac Cardiovasc Surg 2014;**148**:2911-17.

[64] Kasama K, Uchida K, Karube N, Takebayashi S, Imoto K, Masuda M. *Impact of Isolated Cerebral Perfusion Technique for Aortic Arch Aneurysm Repair in Elderly Patients*. Annals of Thoracic Surgery 2019;**107**:533-38.

[65] Kazui T, Yamashita K, Washiyama N, Terada H, Muhammad Bashar AH, Suzuki T *et al.* *Usefulness of antegrade selective cerebral perfusion during aortic arch operations*. Ann Thorac Surg 2002;**74**:S1806-S09.

[66] Kazui T, Washiyama N, Muhammad BAH, Terada H, Yamashita K, Takinami M *et al.* *Extended total arch replacement for acute type A aortic dissection: Experience with seventy patients*. J Thorac Cardiovasc Surg 2000;**119**:558-65.

[67] Khaladj N, Shrestha M, Meck S, Peterss S, Kamiya H, Kallenbach K *et al.* *Hypothermic circulatory arrest with selective antegrade cerebral perfusion in ascending aortic and aortic arch surgery: A risk factor analysis for adverse outcome in 501 patients*. J Thorac Cardiovasc Surg 2008;**135**:908-14.

[68] Khullar V, Schaff HV, Dearani JA, Daly RC, Greason KL, Joyce LD *et al.* *Open Surgical Repair Remains the Gold Standard for Treating Aortic Arch Pathology*. Annals of Thoracic Surgery;**103**:1413-20.

[69] Kim JW, Choi JY, Rhie S, Lee CE, Sim HJ, Park HO. *Clinical Results of Ascending Aorta and Aortic Arch Replacement under Moderate Hypothermia with Right Brachial and Femoral Artery Perfusion*. Korean j thorac cardiovasc surg;**44**:215-19.

[70] Kim JH, Lee SH, Lee S, Youn YN, Yoo KJ, Joo HC. *Axillary artery cannulation reduces early embolic stroke and mortality after open arch repair with circulatory arrest*. J Thorac Cardiovasc Surg 2019.

[71] Kim JH, Choi JB, Kim TY, Kim KH, Kuh JH. *Simplified surgical approach to improve surgical outcomes in the center with a small volume of acute type A aortic dissection surgery*. Technol Health Care 2018;**26**:675-85.

[72] Kirali K, Omeroglu SN, Ardal H, Toker ME, Erdogan HB, Daglar B *et al.* *Long-term comparison of aortic arch replacement with or without elephant trunk procedure via retrograde cerebral perfusion for aortic arch dissection*. Cardiovascular Surgery 2002;**10**:38-44.

[73] Kozlov BN, Panfilov DS, Ponomarenko IV, Miroshnichenko AG, Nenakhova AA, Maksimov AI *et al.* *The risk of spinal cord injury during the frozen elephant trunk procedure in acute aortic dissection*. Interact Cardiovasc Thorac Surg 2018;**26**:972-76.

[74] Kremer J, Preisner F, Dib B, Tochtermann U, Ruhparwar A, Karck M *et al.* *Aortic arch replacement with frozen elephant trunk technique - a single-center study*. J Cardiothorac Surg 2019;**14**:147.

[75] Küçüker Ş A, Özatik MA, Saritaş A, Taşdemir O. *Arch repair with unilateral antegrade cerebral perfusion*. Eur J Cardio-thorac Surg 2005;**27**:638-43.

[76] Kunihara T, Grün T, Aicher D, Langer F, Adam O, Wendler O *et al.* *Hypothermic circulatory arrest is not a risk factor for neurologic morbidity in aortic surgery: A propensity score analysis*. J Thorac Cardiovasc Surg 2005;**130**:712-18.

[77] Lakew F, Pasek P, Zacher M, Diegeler A, Urbanski PP. *Femoral versus aortic cannulation for surgery of chronic ascending aortic aneurysm*. Annals of Thoracic Surgery;**80**:84-89.

[78] Lau C, Gaudino M, Iannacone EM, Gambardella I, Munjal M, Ohmes LB *et al.* *Retrograde Cerebral Perfusion Is Effective for Prolonged Circulatory Arrest in Arch Aneurysm Repair*. Ann Thorac Surg 2018;**105**:491-97.

[79] Lee JH, Chung CH, Kang JK, Choo SJ, Song H, Lee JW. *Unilateral antegrade selective cerebral perfusion in aortic surgery: clinical outcomes at different levels of hypothermia*. J Korean Med Sci 2009;**24**:807-11.

[80] Legras A, Bruzzi M, Nakashima K, Hillion ML, Loisance D, Kirsch M. *Colder is better during hypothermic circulatory arrest for acute type a aortic dissection*. Scandinavian Cardiovascular Journal 2013;**47**:121-28.

[81] Lei Q, Chen L, Jin M, Ji H, Yu Q, Cheng W *et al.* *Preoperative and Intraoperative Risk Factors for Prolonged Intensive Care Unit Stay After Aortic Arch Surgery*. J Cardiothorac Vasc Anesth 2009;**23**:789-94.

[82] Leontyev S, Davierwala PM, Semenov M, von Aspern K, Krog G, Noack T *et al.* *Antegrade selective cerebral perfusion reduced in-hospital mortality and permanent focal neurological deficit in patients with elective aortic arch surgery†*. Eur J Cardiothorac Surg 2019.

[83] Leshnower BG, Myung RJ, Thourani VH, Halkos ME, Kilgo PD, Puskas JD *et al.* *Hemiarch replacement at 28°C: An analysis of mild and moderate hypothermia in 500 patients*. Ann Thorac Surg 2012;**93**:1910-16.

[84] Li B, Hu X, Wang Z. *The neurologic protection of unilateral versus bilateral antegrade cerebral perfusion in aortic arch surgery with deep hypothermic circulatory arrest: A study of 77 cases*. Int J Surg 2017;**40**:8-13.

[85] Liu H, Wang X, Liu S, Cong S, Lu Y, Yang Y *et al.* *Postoperative hypothermia after total aortic arch replacement in acute type A aortic dissection-multivariate analysis and risk identification for postoperative hypothermia occurrence*. J Thorac Dis 2020;**12**:7089-96.

[86] Lopez Almodovar LF, Lima Canadas P, Enriquez Puga A, Narvaez Mayorga I, Buendia Minano JA, Sanchez Casado M *et al.* *Single Low-Volume Center Experience with Frozen Elephant Trunk in Acute Type A Aortic Dissections*. Aorta (Stamford) 2018;**6**:125-29.

[87] Lu S, Sun X, Hong T, Yang S, Song K, Lai H *et al.* *Bilateral versus unilateral antegrade cerebral perfusion in arch reconstruction for aortic dissection*. Ann Thorac Surg 2012;**93**:1917-20.

[88] Lytle BW, McCarthy PM, Meaney KM, Stewart RW, Cosgrove IDM. *Systemic hypothermia and circulatory arrest combined with arterial perfusion of the superior vena cava: Effective intraoperative cerebral protection*. J THORAC CARDIOVASC SURG 1995;**109**:738-43.

[89] Ma M, Liu L, Feng X, Wang Y, Hu M, Pan T *et al.* *Moderate Hypothermic Circulatory Arrest with Antegrade Cerebral Perfusion for Rapid Total Arch Replacement in Acute Type A Aortic Dissection*. Thorac Cardiovasc Surg 2015;**64**:124-32.

[90] Ma H, Xiao Z, Shi J, Liu L, Qin C, Guo Y. *Aortic arch cannulation with the guidance of transesophageal echocardiography for Stanford type A aortic dissection*. J Cardiothorac Surg 2018;**13**:106.

[91] Malaisrie SC, Duncan BF, Mehta CK, Badiwala MV, Rinewalt D, Kruse J *et al.* *The addition of hemiarch replacement to aortic root surgery does not affect safety*. J Thorac Cardiovasc Surg 2015;**150**:118-24.

[92] Malvindi PG, Modi A, Miskolczi S, Kaarne M, Velissaris T, Barlow C *et al.* *Open and closed distal anastomosis for acute type A aortic dissection repair*. Interact Cardiovasc Thorac Surg;**22**:776-83.

[93] Maroto LC, Carnero M, Cobiella J, Beltrao R, Villagrán E, Reguillo F *et al.* *Single-center experience and evolution of technique with the E-vita Open prosthesis*. J Card Surg 2020;**35**:2663-71.

[94] Matalanis G, Hata M, Buxton BF. *A retrospective comparative study of deep hypothermic circulatory arrest, retrograde, and antegrade cerebral perfusion in aortic arch surgery*. Ann Thorac Cardiovasc Surg 2003;**9**:174-79.

[95] Matsuyama S, Tabata M, Shimokawa T, Matsushita A, Fukui T, Takanashi S. *Outcomes of total arch replacement with stepwise distal anastomosis technique and modified perfusion strategy*. J Thorac Cardiovasc Surg 2012;**143**:1377-81.

[96] Matsuzaki Y, Hirayama T, Uesugi H, Ideta I, Oshitomi T. *Direct cannulation of arch vessels for total arch replacement*. Cardiovasc Thorac Open 2017;**3**.

[97] Matt P, Banerjee P, Grapow M, Rueter F, Schurr U, Siegemund M *et al.* *Modified frozen elephant trunk for acute type A aortic dissection: A comparative study with standard repair technique*. Eur J Cardio-thorac Surg 2017;**51**:754-60.

[98] Milewski RK, Pacini D, Moser GW, Moeller P, Cowie D, Szeto WY *et al.* *Retrograde and Antegrade Cerebral Perfusion: Results in Short Elective Arch Reconstructive Times*. Ann Thorac Surg 2010;**89**:1448-57.

[99] Minakawa M, Fukuda I, Yamauchi S, Watanabe K, Kawamura T, Taniguchi S *et al.* *Early and Long-Term Outcome of Total Arch Replacement Using Selective Cerebral Perfusion*. Annals of Thoracic Surgery 2010;**90**:72-77.

[100] Miyamoto S, Takahashi S, Okahara S, Takahashi H, Katayama K, Watanabe M *et al.* *Abdominal organ protection strategy for aortic arch aneurysm surgery*. Perfusion 2018;**33**:512-19.

[101] Mori Y, Hirose H, Takagi H, Umeda Y, Fukumoto Y, Shimabukuro K *et al.* *Aortic arch repair for Stanford type A aortic dissection with distal anastomosis to the proximal level of the distal aortic arch*. J Thorac Cardiovasc Surg 2003;**126**:415-19.

[102] Murzi M, Miceli A, Di Stefano G, Cerillo AG, Kallushi E, Farneti P *et al.* *Enhancing quality control and performance monitoring in thoracic aortic surgery: A 10-year single institutional experience*. Eur J Cardio-thorac Surg 2015;**47**:608-15.

[103] Nakamura K, Onitsuka T, Yano M, Yano Y, Saitoh T, Kojima K *et al.* *Predictor of neurologic dysfunction after elective thoracic aorta repair using selective cerebral perfusion*. Scandinavian Cardiovascular Journal;**39**:96-101.

[104] Nakamura K, Nakamura E, Yano M, Niina K, Kojima K, Onitsuka T. *Factors influencing permanent neurologic dysfunction and mortality after total arch replacement with separate arch vessel grafting using selective cerebral perfusion*. Ann Thorac Cardiovasc Surg 2011;**17**:39-44.

[105] Nakamura T, Mikamo A, Matsuno Y, Fujita A, Kurazumi H, Suzuki R *et al.* *Impact of acute kidney injury on prognosis of chronic kidney disease after aortic arch surgery*. Interact Cardiovasc Thorac Surg 2020;**30**:273-79.

[106] Numata S, Tsutsumi Y, Monta O, Yamazaki S, Seo H, Yoshida S *et al.* *Mid-Long-Term Results After Aortic Arch Repair Using a Four-Branched Graft With Antegrade Selective Cerebral Perfusion*. Journal of Cardiac Surgery 2013;**28**:537-42.

[107] Numata S, Thomson DS, Seah P, Singh T. *Simplified Cerebral Protection Using Unilateral Antegrade Cerebral Perfusion and Moderate Hypothermic Circulatory Arrest*. Heart Lung Circul 2009;**18**:334-36.

[108] Numata S, Ogino H, Sasaki H, Hanafusa Y, Hirata M, Ando M *et al.* *Total arch replacement using antegrade selective cerebral perfusion with right axillary artery perfusion*. European Journal of Cardio-Thoracic Surgery 2003;**23**:771-75.

[109] Ochiai Y, Imoto Y, Sakamoto M, Ueno Y, Sano T, Baba H *et al.* *Long-term effectiveness of total arch replacement for type A aortic dissection*. Ann Thorac Surg 2005;**80**:1297-302.

[110] Ogino H, Sasaki H, Minatoya K, Matsuda H, Tanaka H, Watanuki H *et al.* *Evolving arch surgery using integrated antegrade selective cerebral perfusion: Impact of axillary artery perfusion*. J Thorac Cardiovasc Surg 2008;**136**:641-49.

[111] Ogino H, Ueda Y, Sugita T, Sakakibara Y, Matsuyama K, Matsubayashi K *et al.* *Surgery for acute type A aortic dissection using retrograde cerebral perfusion*. Jpn J Thorac Cardiovasc Surg 2001;**49**:337-42.

[112] Ohata T, Sakakibara T, Takano H, Ishizaka T. *Total arch replacement for thoracic aortic aneurysm via median sternotomy with or without left anterolateral thoracotomy*. Ann Thorac Surg 2003;**75**:1792-96.

[113] Ohtsubo S, Itoh T, Takarabe K, Rikitake K, Furukawa K, Suda H *et al.* *Surgical results of hemiarch replacement for acute type A dissection*. Ann Thorac Surg 2002;**74**:S1853-S56.

[114] Okada T, Shimamoto M, Yamazaki F, Nakai M, Miura Y, Itonaga T *et al.* *Insights of stroke in aortic arch surgery: Identification of significant risk factors and surgical implication*. Gen Thorac Cardiovasc Surg 2012;**60**:268-74.

[115] Okita Y, Okada K, Omura A, Kano H, Minami H, Inoue T *et al.* *Total arch replacement using antegrade cerebral perfusion*. Journal of Thoracic and Cardiovascular Surgery 2013;**145**:S63-S71.

[116] Olsson C, Thelin S. *Antegrade cerebral perfusion with a simplified technique: Unilateral versus bilateral perfusion*. Ann Thorac Surg 2006;**81**:868-74.

[117] Orlov CP, Orlov OI, Shah VN, Kilcoyne M, Buckley M, Sicouri S *et al.* *Total Arch Replacement with Hypothermic Circulatory Arrest, Antegrade Cerebral Perfusion and the Y-graft*. Semin Thorac Cardiovasc Surg 2020;**32**:683-91.

[118] Ozerdem G, Ozdemir O, Kaya B. *Direct cannulation of axillary artery via transpectoral approach in ascending aorta and aortic arch surgery*. Turk Gogus Kalp Damar Cerrahisi Dergisi-Turkish Journal of Thoracic and Cardiovascular Surgery;**19**:503-06.

[119] Pacini D, Pantaleo A, Di Marco L, Leone A, Barberio G, Parolari A *et al.* *Risk factors for acute kidney injury after surgery of the thoracic aorta using antegrade selective cerebral perfusion and moderate hypothermia*. Journal of Thoracic and Cardiovascular Surgery 2015;**150**:127-+.

[120] Pagni S, Ganzel BL, Trivedi JR, Singh R, Mascio CE, Austin EH *et al.* *Early and midterm outcomes following surgery for acute type A aortic dissection*. J Card Surg 2013;**28**:543-49.

[121] Panos A, Murith N, Bednarkiewicz M, Khatchatourov G. *Axillary cerebral perfusion for arch surgery in acute type A dissection under moderate hypothermia*. Eur J Cardio-thorac Surg 2006;**29**:1036-39.

[122] Park SJ, Jeon BB, Kim HJ, Kim JB. *Aortic arch repair under moderate hypothermic circulatory arrest with or without antegrade cerebral perfusion based on the extent of repair*. J Thorac Dis 2018;**10**:1875-83.

[123] Patel HJ, Nguyen C, Diener AC, Passow MC, Salata D, Deeb GM. *Open arch reconstruction in the endovascular era: Analysis of 721 patients over 17 years*. J Thorac Cardiovasc Surg 2011;**141**:1417-23.

[124] Perera NK, Shi WY, Koirala RS, Galvin SD, McCall PR, Matalanis G. *Outcomes of Aortic Arch Replacement Performed Without Circulatory Arrest or Deep Hypothermia*. Aorta (Stamford);**1**:102-09.

[125] Perreas K, Samanidis G, Thanopoulos A, Georgiopoulos G, Antoniou T, Khoury M *et al.* *Antegrade or retrograde cerebral perfusion in ascending aorta and hemiarch surgery? a propensity-matched analysis*. Ann Thorac Surg 2016;**101**:146-52.

[126] Preventza O, Price MD, Spiliotopoulos K, Amarasekara HS, Cornwell LD, Omer S *et al.* *In elective arch surgery with circulatory arrest, does the arterial cannulation site really matter? A propensity score analysis of right axillary and innominate artery cannulation*. J Thorac Cardiovasc Surg 2018;**155**:1953-60.e4.

[127] Qian H, Hu J, Du L, Xue Y, Meng W, Zhang EY. *Modified hypothermic circulatory arrest for emergent repair of acute aortic dissection type a: a single-center experience*. J Cardiothorac Surg;**8**.

[128] Rungatscher A, Luciani GB, Linardi D, Milani E, Gottin L, Walpoth B *et al.* *Temperature Variation after Rewarming from Deep Hypothermic Circulatory Arrest Is Associated with Survival and Neurologic Outcome*. Ther Hypothermia Temp Manage 2017;**7**:101-06.

[129] Salem M, Friedrich C, Thiem A, Salem MA, Erdal Y, Puehler T *et al.* *Influence of moderate hypothermic circulatory arrest on outcome in patients undergoing elective replacement of thoracic aorta*. J Thorac Dis 2020;**12**:5756-64.

[130] Salem M, Friedrich C, Rusch R, Frank D, Hoffmann G, Lutter G *et al.* *Is total arch replacement associated with an increased risk after acute type A dissection?* J Thorac Dis 2020;**12**:5517-31.

[131] Samanidis G, Katselis C, Contrafouris C, Georgiopoulos G, Kriaras I, Antoniou T *et al.* *Predictors of Outcomes after Correction of Acute Type A Aortic Dissection under Moderate Hypothermic Circulatory Arrest and Antegrade Cerebral Perfusion*. Braz J Cardiovasc Surg 2018;**33**:143-50.

[132] Schneider SRB, Dell'Aquila AM, Akil A, Schlarb D, Panuccio G, Martens S *et al.* *Results of "elephant trunk" total aortic arch replacement using a multi-branched, collared graft prosthesis*. Heart Vessels;**31**:390-96.

[133] Shimamura K, Kuratani T, Matsumiya G, Kato M, Shirakawa Y, Takano H *et al.* *Long-term results of the open stent-grafting technique for extended aortic arch disease*. J Thorac Cardiovasc Surg 2008;**135**:1261-69.

[134] Shimazaki Y, Watanabe T, Takahashi T, Minowa T, Inui K, Uchida T *et al.* *Minimized mortality and neurological complications in surgery for chronic arch aneurysm: Axillary artery cannulation, selective cerebral perfusion, and replacement of the ascending and total arch aorta*. J Card Surg 2004;**19**:338-42.

[135] Shimizu H, Matayoshi T, Morita M, Ueda T, Yozu R. *Total arch replacement under flow monitoring during selective cerebral perfusion using a single pump*. Ann Thorac Surg 2013;**95**:29-34.

[136] Shrestha M, Martens A, Behrendt S, Maeding I, Koigeldiyev N, Haverich A. *Is the branched graft technique better than the en bloc technique for total aortic arch replacement?* Eur J Cardio-thorac Surg 2014;**45**:181-87.

[137] Sinatra R, Melina G, Pulitani I, Fiorani B, Ruvolo G, Marino B. *Emergency operation for acute type A aortic dissection: Neurologic complications and early mortality*. Ann Thorac Surg 2001;**71**:33-38.

[138] Spielvogel D, Etz CD, Silovitz D, Lansman SL, Griepp RB. *Aortic Arch Replacement With a Trifurcated Graft*. Ann Thorac Surg 2007;**83**:S791-S95.

[139] Stowe CL, Baertlein MA, Wierman MD, Rucker M, Ebra G. *Surgical management of ascending and aortic arch disease: Refined techniques with improved results*. Ann Thorac Surg 1998;**66**:388-95.

[140] Suda H, Itoh T, Natsuaki M, Minato N, Ueno T, Ohteki H. *Surgical treatment for acute aortic arch dissection*. Cardiovasc surg 1996;**4**:315-19.

[141] Sultan I, Bianco V, Yazji I, Kilic A, Dufendach K, Cardounel A *et al.* *Hemiarch Reconstruction Versus Clamped Aortic Anastomosis for Concomitant Ascending Aortic Aneurysm*. Ann Thorac Surg 2018;**106**:750-56.

[142] Sundt ITM, Orszulak TA, Cook DJ, Schaff HV. *Improving Results of Open Arch Replacement*. Ann Thorac Surg 2008;**86**:787-96.

[143] Sundt TM, Moon MR, DeOliviera N, McDonald J, Camillo CJ, Pasque MK. *Contemporary results of total aortic arch replacement*. Journal of Cardiac Surgery;**19**:235-39.

[144] Svensson LG, Crawford ES, Hess KR, Coselli JS, Raskin S, Shenaq SA *et al.* *Deep hypothermia with circulatory arrest: Determinants of stroke and early mortality in 656 patients*. J thorac cardiovasc surg 1993;**106**:19-31.

[145] Tabayashi K, Niibori K, Iguchi A, Shoji Y, Ohmi M, Mohri H. *Replacement of the transverse aortic arch for type A acute aortic dissection*. Ann thorac surg 1993;**55**:864-67.

[146] Takahara Y, Mogi K, Sakurai M, Nishida H. *Total Aortic Arch Grafting via Median Sternotomy Using Integrated Antegrade Cerebral Perfusion*. Ann Thorac Surg 2003;**76**:1485-89.

[147] Takashima N, Suzuki T, Asai T, Nota H, Ikegami H, Kinoshita T *et al.* *Outcome of total arch replacement with coronary artery bypass grafting*. Eur J Cardio-thorac Surg 2015;**47**:990-94.

[148] Takayama H, Smith CR, Bowdish ME, Stewart AS. *Open distal anastomosis in aortic root replacement using axillary cannulation and moderate hypothermia*. J Thorac Cardiovasc Surg 2009;**137**:1450-53.

[149] Tan PS, Aveling W, Pugsley WB, Newman SP, Treasure T. *Experience with circulatory arrest and hypothermia to facilitate thoracic aortic surgery*. Ann R Coll Surg Engl;**71**:81-86.

[150] Tang GHL, Malekan R, Yu CJ, Kai M, Lansman SL, Spielvogel D. *Surgery for acute type A aortic dissection in octogenarians is justified*. J Thorac Cardiovasc Surg 2013;**145**:S186-S90.

[151] Taniguchi K, Toda K, Hata H, Shudo Y, Matsue H, Takahashi T *et al.* *Elephant Trunk Anastomosis Proximal to Origin of Innominate Artery in Total Arch Replacement*. Ann Thorac Surg 2007;**84**:1729-34.

[152] Tokuda Y, Fujimoto K, Narita Y, Mutsuga M, Terazawa S, Ito H *et al.* *Spinal cord injury following aortic arch replacement*. Surg Today 2020;**50**:106-13.

[153] Tong G, Zhang B, Zhou X, Tao Y, Yan T, Wang X *et al.* *Bilateral versus unilateral antegrade cerebral perfusion in total arch replacement for type A aortic dissection*. J Thorac Cardiovasc Surg 2017;**154**:767-75.

[154] Toyama M, Matsumura Y, Tamenishi A, Okamoto H. *Safety of mild hypothermic circulatory arrest with selective cerebral perfusion*. Asian Cardiovasc Thorac Ann 2009;**17**:500-04.

[155] Tsagakis K, Pacini D, Di Bartolomeo R, Gorlitzer M, Weiss G, Grabenwoger M *et al.* *Multicenter early experience with extended aortic repair in acute aortic dissection: is simultaneous descending stent grafting justified?* J Thorac Cardiovasc Surg;**140**:S116-20; discussion S42-S46.

[156] Uchida N, Katayama A, Tamura K, Sutoh M, Kuraoka M, Murao N *et al.* *Long-term results of the frozen elephant trunk technique for extended aortic arch disease*. Eur J Cardio-thorac Surg 2010;**37**:1338-45.

[157] Ueda T, Shimizu H, Hashizume K, Koizumi K, Mori M, Shin H *et al.* *Mortality and morbidity after total arch replacement using a branched arch graft with selective antegrade cerebral perfusion*. Annals of Thoracic Surgery 2003;**76**:1951-56.

[158] Unal M, Yilmaz O, Akar I, Ince I, Aslan C, Koc F *et al.* *Brachiocephalic Artery Cannulation in Proximal Aortic Surgery that Requires Circulatory Arrest*. Tex Heart Inst J;**41**:596-600.

[159] Urbanski PP, Thamm T, Bougioukakis P, Irimie V, Prasad P, Diegeler A *et al.* *Efficacy of unilateral cerebral perfusion for brain protection in aortic arch surgery*. J Thorac Cardiovasc Surg 2019.

[160] Usui A, Yasuura K, Watanabe T, Maseki T. *Comparative clinical study between retrograde cerebral perfusion and selective cerebral perfusion in surgery for acute type A aortic dissection*. European Journal of Cardio-Thoracic Surgery 1999;**15**:571-78.

[161] Wai Sang SL, Beute TJ, Timek T. *A simple method to establish antegrade cerebral perfusion during hemiarch reconstruction*. JTCVS Tech 2020;**2**:10-15.

[162] Wang X, Yang F, Zhu J, Liu Y, Sun L, Hou X. *Aortic arch surgery with hypothermic circulatory arrest and unilateral antegrade cerebral perfusion: Perioperative outcomes*. J Thorac Cardiovasc Surg 2019.

[163] Watanabe G, Ohtake H, Tomita S, Yamaguchi S, Kimura K, Yashiki N. *Tepid hypothermic (32°c) circulatory arrest for total aortic arch replacement: A paradigm shift from profound hypothermic surgery*. Interact Cardiovasc Thorac Surg 2011;**12**:952-55.

[164] Wiedemann D, Kocher A, Dorfmeister M, Vadehra A, Mahr S, Laufer G *et al.* *Effect of cerebral protection strategy on outcome of patients with Stanford type A aortic dissection*. J Thorac Cardiovasc Surg 2013;**146**:647-55.e1.

[165] Wu Y, Xiao L, Yang T, Wang L, Chen X. *Aortic arch reconstruction: Deep and moderate hypothermic circulatory arrest with selective antegrade cerebral perfusion*. Perfusion 2017;**32**:389-93.

[166] Wu Y, Jiang R, Li Z, Pan Y, Yang L, Wang T *et al.* *Application of a Modified Extracorporeal Circulation Perfusion Method During Surgery for Acute Stanford Type A Aortic Dissection*. Heart Lung Circul 2020;**29**:1203-09.

[167] Yamamoto N, Nie M, Hari Y, Tanaka Y, Ohara K, Miyaji K. *A selection of cases of direct cannulation in surgery for type A dissection*. Asian Cardiovasc Thorac Ann 2014;**22**:284-87.

[168] Yilmazkaya B, Gurkahraman S, Yondem OZ, Hijazi A, Algin IH, Yesilay A. *Advantages of upper brachial artery cannulation in aortic surgery*. Asian Cardiovasc Thorac Ann 2014;**22**:18-24.

[169] Yu A, Zhang M, Wang Z, Hu Z, Hu R, Xiao Q. *Elephant trunk stent fenestration for acute type A aortic dissection*. Ann Thorac Surg 2020.

[170] Zheng J, Xu SD, Zhang YC, Zhu K, Gao HQ, Zhang K *et al.* *Association between cardiopulmonary bypass time and 90-day post-operative mortality in patients undergoing arch replacement with the frozen elephant trunk: A retrospective cohort study*. Chin Med J 2019;**132**:2325-32.

[171] Zierer A, Moritz A. *Cerebral Protection for Aortic Arch Surgery: Mild Hypothermia With Selective Cerebral Perfusion*. Semin Thorac Cardiovasc Surg 2012;**24**:123-26.

[172] Zierer A, Risteski P, El-Sayed Ahmad A, Moritz A, Diegeler A, Urbanski PP. *The impact of unilateral versus bilateral antegrade cerebral perfusion on surgical outcomes after aortic arch replacement: A propensity-matched analysis*. J Thorac Cardiovasc Surg 2014;**147**:1212-18.

[173] Zierer A, Moon MR, Melby SJ, Moazami N, Lawton JS, Kouchoukos NT *et al.* *Impact of perfusion strategy on neurologic recovery in acute type A aortic dissection*. Ann Thorac Surg 2007;**83**:2122-28; discussion 28-29.

[174] Shihata M, Mittal R, Senthilselvan A, Ross D, Koshal A, Mullen J *et al.* *Selective antegrade cerebral perfusion during aortic arch surgery confers survival and neuroprotective advantages*. J Thorac Cardiovasc Surg 2011;**141**:948-52.

[175] Preventza O, Coselli JS, Price MD, Simpson KH, Yafei O, Cruz KDL *et al.* *Elective primary aortic root replacement with and without hemiarch repair in patients with no previous cardiac surgery*. J Thorac Cardiovasc Surg 2017;**153**:1402-08.

[176] Misfeld M, Leontyev S, Borger MA, Gindensperger O, Lehmann S, Legare JF *et al.* *What is the best strategy for brain protection in patients undergoing aortic arch surgery? A single center experience of 636 patients*. Ann Thorac Surg 2012;**93**:1502-08.

[177] Tarola CL, Losenno KL, Gelinas JJ, Jones PM, Fernandes P, Fox SA *et al.* *Whole body perfusion strategy for aortic arch repair under moderate hypothermia*. Perfusion 2018;**33**:254-63.

[178] Norton EL, Wu XT, Kim KM, Patel HJ, Deeb GM, Yang B. *Unilateral is comparable to bilateral antegrade cerebral perfusion in acute type A aortic dissection repair*. Journal of Thoracic and Cardiovascular Surgery 2020;**160**:617-+.

[179] Peterson MD, Garg V, Mazer CD, Chu MWA, Bozinovski J, Dagenais F *et al.* *A randomized trial comparing axillary versus innominate artery cannulation for aortic arch surgery*. J Thorac Cardiovasc Surg 2021.

[180] Strauch JT, Spielvogel D, Lauten A, Galla JD, Lansman SL, McMurtry K *et al.* *Technical advances in total aortic arch replacement*. Ann Thorac Surg 2004;**77**:581-90.

[181] Zhang K, Pan XD, Dong SB, Zheng J, Xu SD, Liu YM *et al.* *Cardiopulmonary bypass duration is an independent predictor of adverse outcome in surgical repair for acute type A aortic dissection*. J Int Med Res 2020;**48**.

[182] Harrington DK, Walker AS, Kaukuntla H, Bracewell RM, Clutton-Brock TH, Faroqui M *et al.* *Selective antegrade cerebral perfusion attenuates brain metabolic deficit in aortic arch surgery: A prospective randomized trial*. Circulation 2004;**110**:II231-II36.

[183] Mourad F, Srivastava V, Duncan A. *Aortic arch surgery using selective antegrade cerebral perfusion and mild hypothermia*. J Egypt Soc Cardio-Thorac Surg 2016;**24**:116-22.

[184] Preventza O, Price MD, Simpson KH, Cooley DA, Pocock E, De La Cruz KI *et al.* *Hemiarch and total arch surgery in patients with previous repair of acute type i aortic dissection*. Ann Thorac Surg 2015;**100**:833-38.

[185] Chu MWA, Losenno KL, Dubois LA, Jones PM, Ouzounian M, Whitlock R *et al.* *Early Clinical Outcomes of Hybrid Arch Frozen Elephant Trunk Repair With the Thoraflex Hybrid Graft*. Ann Thorac Surg 2019;**107**:47-53.

[186] Della Corte A, Scardone M, Romano G, Amarelli C, Biondi A, De Santo LS *et al.* *Aortic arch surgery: Thoracoabdominal perfusion during antegrade cerebral perfusion may reduce postoperative morbidity*. Ann Thorac Surg 2006;**81**:1358-64.

[187] Okita Y, Minatoya K, Tagusari O, Ando M, Nagatsuka K, Kitamura S. *Prospective comparative study of brain protection in total aortic arch replacement: Deep hypothermic circulatory arrest with retrograde cerebral perfusion or selective antegrade cerebral perfusion*. Ann Thorac Surg 2001;**72**:72-79.

[188] Hata M, Suzuki M, Sezai A, Niino T, Yoshitake I, Unosawa S *et al.* *Outcome of less invasive proximal arch replacement with moderate hypothermic circulatory arrest followed by aggressive rapid re-warming in emergency surgery for type A acute aortic dissection*. Circ J 2009;**73**:69-72.

[189] Perreas K, Samanidis G, Dimitriou S, Kalogris P, Balanika M, Antzaka C *et al.* *Outcomes after ascending aorta and proximal aortic arch repair using deep hypothermic circulatory arrest with retrograde cerebral perfusion: Analysis of 207 patients*. Interact Cardiovasc Thorac Surg 2012;**15**:456-61.

[190] Müller D, Fieguth HG, Wimmer-Greinecker G. *Neurologic outcome after surgery of the aortic arch: Comparison of deep hypothermic arrest, antegrade and retrograde cerebral perfusion*. Indian Journal of … 2004.

[191] Estrera AL, Miller CC, Lee TY, Shah P, Irani AD, Ganim N *et al.* *Integrated cerebral perfusion for hypothermic circulatory arrest during transverse aortic arch repairs*. Eur J Cardio-thorac Surg 2010;**38**:293-98.

[192] Yaǧdl T, Atay Y, Çikirikçioǧlu M, Boǧa M, Posacioǧlu H, Özbaran M *et al.* *Determinants of early mortality and neurological morbidity in aortic operations performed under circulatory arrest*. J Card Surg 2000;**15**:186-93.

[193] Moshkovitz Y, David TE, Caleb M, Feindel CM, De Sa MPL. *Circulatory arrest under moderate systemic hypothermia and cold retrograde cerebral perfusion*. Ann Thorac Surg 1998;**66**:1179-84.

[194] Urbanski PP, Siebel A, Zacher M, Hacker RW. *Is extended aortic replacement in acute type A dissection justifiable?* Ann Thorac Surg 2003;**75**:525-29.

[195] Ehrlich MP, Ergin MA, McCullough JN, Lansman SL, Galla JD, Bodian CA *et al.* *Results of immediate surgical treatment of all acute type A dissections*. Circulation 2000;**102**:III248-III52.

[196] Stamou SC, Rausch LA, Kouchoukos NT, Lobdell KW, Khabbaz K, Murphy E *et al.* *Comparison between antegrade and retrograde cerebral perfusion or profound hypothermia as brain protection strategies during repair of type A aortic dissection*. Ann Cardiothorac Surg 2016;**5**:328-35.

[197] Di Eusanio M, Schepens M, Morshuis WJ, Dossche KM, Di Bartolomeo R, Pacini D *et al.* *Brain protection using antegrade selective cerebral perfusion: A multicenter study*. Ann Thorac Surg 2003;**76**:1181-88.

[198] Kondo N, Tamura K, Hiraoka A, Totsugawa T, Chikazawa G, Sakaguchi T *et al.* *Treatment outcomes for acute type A aortic dissection with patent false lumen in patients over the age of 80*. General Thoracic and Cardiovascular Surgery 2019;**67**:765-72.

[199] Inoue Y, Minatoya K, Oda T, Itonaga T, Seike Y, Tanaka H *et al.* *Surgical outcomes for acute type A aortic dissection with aggressive primary entry resection*. Eur J Cardio-thorac Surg 2016;**50**:567-73.

[200] Bavaria JE, Brinster DR, Gorman RC, Woo YJ, Gleason T, Pochettino A. *Advances in the treatment of acute type A dissection: An integrated approach*. Ann Thorac Surg 2002;**74**:S1848-S52.

[201] Vallabhajosyula P, Jassar AS, Menon RS, Komlo C, Gutsche J, Desai ND *et al.* *Moderate versus deep hypothermic circulatory arrest for elective aortic transverse hemiarch reconstruction*. Ann Thorac Surg 2015;**99**:1511-17.

[202] Luo HY, Hu KJ, Zhou JY, Wang CS. *Analysis of the risk factors of postoperative respiratory dysfunction of type A aortic dissection and lung protection*. Perfusion-Uk;**24**:199-202.

[203] Algarni KD, Yanagawa B, Rao V, Yau TM. *Profound hypothermia compared with moderate hypothermia in repair of acute type A aortic dissection*. Journal of Thoracic and Cardiovascular Surgery 2014;**148**:2888-94.

[204] Saritas A, Kervan U, Vural KM, Kucuker SA, Yavas S, Birincioglu LC. *Visceral protection during moderately hypothermic selective antegrade cerebral perfusion through right brachial artery*. Eur J Cardio-thorac Surg 2010;**37**:669-76.

[205] Leshnower BG, Myung RJ, Chen EP. *Aortic arch surgery using moderate hypothermia and unilateral selective antegrade cerebral perfusion*. Ann cardiothorac surg 2013;**2**:288-95.

[206] Ma WG, Zheng J, Zhang W, Sun K, Ziganshin BA, Wang LF *et al.* *Frozen elephant trunk with total arch replacement for type A aortic dissections: Does acuity affect operative mortality?* J Thorac Cardiovasc Surg 2014;**148**:963-72.

[207] Czerny M, König T, Reineke D, Sodeck GH, Rieger M, Schoenhoff F *et al.* *Total surgical aortic arch replacement as a safe strategy to treat complex multisegmental proximal thoracic aortic pathology*. Interact Cardiovasc Thorac Surg 2013;**17**:532-36.

[208] Di Eusanio M, Borger M, Petridis FD, Leontyev S, Pantaleo A, Moz M *et al.* *Conventional versus frozen elephant trunk surgery for extensive disease of the thoracic aorta*. J Cardiovasc Med 2014;**15**:803-09.

[209] Beckmann E, Martens A, Kaufeld T, Natanov R, Krueger H, Haverich A *et al.* *Is total aortic arch replacement with the frozen elephant trunk procedure reasonable in elderly patients?* 2021.

[210] Okada K, Omura A, Kano H, Nakai H, Miyahara S, Minami H *et al.* *Outcome of elective total aortic arch replacement in patients with non-dialysis-dependent renal insufficiency stratified by estimated glomerular filtration rate*. Journal of Thoracic and Cardiovascular Surgery;**147**:966-+.

[211] Chu MWA, Losenno KL, Gelinas JJ, Garg V, Dickson J, Harrington A *et al.* *Innominate and Axillary Cannulation in Aortic Arch Surgery Provide Similar Neuroprotection*. Can J Cardiol 2016;**32**:117-23.

[212] Spielvogel D, Halstead JC, Meier M, Kadir I, Lansman SL, Shahani R *et al.* *Aortic arch replacement using a trifurcated graft: Simple, versatile, and safe*. Ann Thorac Surg 2005;**80**:90-95.

[213] Iba Y, Minatoya K, Matsuda H, Sasaki H, Tanaka H, Oda T *et al.* *How should aortic arch aneurysms be treated in the endovascular aortic repair era? A risk-adjusted comparison between open and hybrid arch repair using propensity score-matching analysis(aEuro)*. European Journal of Cardio-Thoracic Surgery;**46**:32-39.

[214] Garg V, Tsirigotis DN, Dickson J, Dalamagas C, Latter DA, Verma S *et al.* *Direct innominate artery cannulation for selective antegrade cerebral perfusion during deep hypothermic circulatory arrest in aortic surgery*. J Thorac Cardiovasc Surg 2014.
